# USP14 and UCHL5 synergistically deubiquitinate PKCα and translocate NF-κB to promote the progression of anaplastic thyroid cancer

**DOI:** 10.1038/s41419-025-07890-9

**Published:** 2025-08-13

**Authors:** Song Zhang, Bing Han, Bo Jiang, Mengyu Chen, Deyang Mu, Qi Wang, Shu Sun, Tong Xu, Feifeng Song, Xinxin Ren, Zongfu Pan, Ping Huang, Minghua Ge

**Affiliations:** 1 Center for Clinical Pharmacy, Cancer Center, Department of Pharmacy, Zhejiang Provincial People’s Hospital (Affiliated People’s Hospital), Hangzhou Medical College, Hangzhou, 310014 China; 2 Zhejiang Key Laboratory of Precision Medicine Research on Head & Neck Cancer, Hangzhou, 310014 China; 3 Department of Pathology, Cancer Center, Zhejiang Provincial People’s Hospital (Affiliated People’s Hospital), Hangzhou Medical College, Hangzhou, 310014 China; 4 Clinical Research Institute, Zhejiang Provincial People’s Hospital (Affiliated People’s Hospital), Hangzhou Medical College, Hangzhou, 310014 China; 5 College of Pharmaceutical Sciences, Hangzhou Medical College, Hangzhou, 310014 China; 6 Department of Head and Neck Surgery, Otolaryngology & Head and Neck Center, Cancer Center, Zhejiang Provincial People’s Hospital (Affiliated People’s Hospital), Hangzhou Medical College, Hangzhou, 310014 China; 7 Zhejiang Provincial Clinical Research Center for Head & Neck Cancer, Hangzhou, 310014 China

**Keywords:** Thyroid diseases, Oncogenes, Ubiquitylation

## Abstract

Anaplastic thyroid carcinoma (ATC), an exceptionally aggressive and rare subtype of thyroid cancer, accounts for 1–2% of all thyroid cancers yet carries a high mortality rate, with a median survival time of less than one year. Despite significant advancements in in the field of thyroid cancer research, effective therapeutic options for ATC remain notably limited. Recently, targeting deubiquitinating enzymes (DUBs) has emerged as a promising strategy in cancer therapy. In this study, we investigated the roles of two DUBs, USP14 and UCHL5, in the progression of ATC. Our findings revealed that both USP14 and UCHL5 were upregulated at both mRNA and protein levels in ATC. Individually silencing USP14 or UCHL5 significantly inhibited the malignant characteristics of ATC, while the simultaneous knockdown of both DUBs proved to be even more efficacious. Furthermore, b-AP15, a dual-targeting inhibitor acting on USP14 and UCHL5, effectively suppressed tumor growth in nude mice. Mechanistically, USP14 and UCHL5 cooperate to stabilize PKCα by concurrently removing K48-linked ubiquitination chains from PKCα, thereby facilitating the nuclear translocation of transcription factor NF-κB and activating the expression of pro-oncogenic and anti-apoptotic genes, such as C-MYC and BCL-XL. These findings suggest that targeting the USP14/UCHL5-PKCα-NF-κB axis may represent a novel therapeutic approach for ATC, offering promising prospects for the development of innovative treatment strategies against this highly lethal disease.

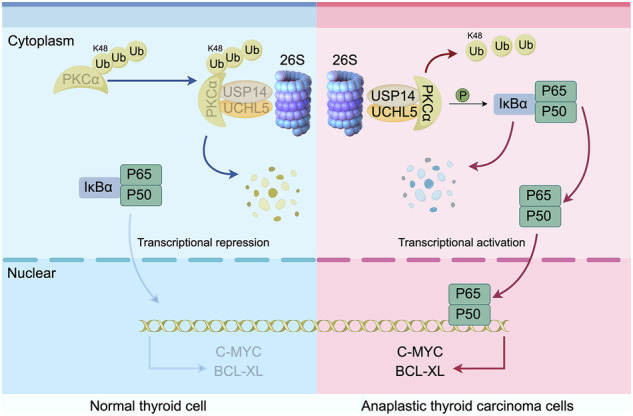

## Introduction

Thyroid cancer, the most common type of endocrine malignancy, has experienced a consistent rise in its incidence over recent years [1, 2]. Anaplastic thyroid carcinoma (ATC), a rare and highly aggressive form of thyroid cancer, comprises merely a small percentage of all thyroid cancers but exhibits an exceptionally high mortality rate with a median survival time of only several months [3–5]. The current clinical approach to treating ATC mainly includes surgical resection, radiation therapy, and chemotherapy [4]. Nevertheless, due to the extremely aggressive characteristics of ATC, these therapeutic options frequently yield less than satisfactory results [4]. Therefore, understanding the mechanisms responsible for the pathogenesis of ATC and identifying effective therapeutic strategies are of critical importance [6].

The ubiquitin-proteasome system (UPS) plays a vital role in the degradation of proteins inside cells and is responsible for regulating the lifecycle of proteins, including synthesis, modification, localization, and degradation [7, 8]. This system is fundamental for maintaining cellular homeostasis, regulating cell cycle, and responding to external stimuli [7, 8]. Deubiquitinating enzymes (DUBs) are a category of proteins specialized in the removal of ubiquitin chains or single ubiquitin molecule from proteins [9]. By reversing ubiquitination, DUBs perform critical regulatory functions within the UPS, restoring protein function and preventing their degradation by the proteasome. Emerging evidence has shown that abnormal DUB expression is closely associated with tumorigenesis and cancer progression [9]. Some DUBs, such as USP7, USP36, and USP13, function to stabilize key oncogenic proteins by preventing their proteasomal degradation, thereby facilitating tumor growth [10–12] Consequently, DUB inhibitors may serve as effective therapeutic agents for cancer [13, 14]. However, non-specific DUB inhibitors have been associated with significant side effects in previous studies, underscoring the necessity for developing specific DUB inhibitors to mitigate these adverse effects [15, 16].

USP14 and UCHL5, members of the DUB family, have been observed to be upregulated in various cancers, playing significant roles in tumor progression [17–20]. b-AP15, a dual-target inhibitor of USP14 and UCHL5 [13], has been shown to be effective in inhibiting the progression of multiple myeloma and in overcoming bortezomib resistance during treatment [21]. It has also demonstrated promising therapeutic effects in esophageal cancer, head and neck cancer, and colorectal cancer [19, 22, 23]. However, there are no reports on the application of b-AP15 in ATC, nor are there studies exploring the roles of USP14 and UCHL5 in this disease. Therefore, it is worthwhile investigating whether b-AP15 exhibits anti-tumor effects in ATC.

In the present study, we conducted a comprehensive investigation to elucidate the roles of USP14 and UCHL5 in the pathogenesis and progression of ATC, with the aim of uncovering their potential as therapeutic targets in this highly aggressive malignancy. Our findings revealed that both USP14 and UCHL5 are upregulated in ATC cells and tissues. Knockdown of either USP14 or UCHL5 significantly inhibits ATC cell proliferation and metastasis, while simultaneous knockdown of both DUBs exhibits even greater efficacy. Furthermore, b-AP15, a dual-targeting inhibitor of USP14 and UCHL5, promotes cell apoptosis and induces cell cycle arrest at the G2/M phase, as well as effectively suppresses tumor growth in nude mice. Mechanistically, USP14 and UCHL5 work synergistically to stabilize PKCα by removing K48-linked ubiquitination chains, leading to the nuclear translocation of the transcription factor NF-κB and the activation of pro-oncogenic and anti-apoptotic signaling pathways. These findings suggested that targeting the USP14/UCHL5-PKCα-NF-κB axis may provide a novel therapeutic approach for ATC, holding significant promise for the development of new and effective treatments against ATC.

## Materials and Methods

### Cell culture

The thyroid cell lines Nthy ori 3-1 (Fuheng Biotechnology), TPC-1 (Procell), BCPAP (Procell), 8505 C (Fuheng Biotechnology), C643 (DSMZ), CAL62 (DSMZ), ARO (Fenghui Biotechnology), and KHM5M (Procell) were cultured in RPMI-1640 medium with 10% FBS at 37 °C in 5% CO_2_. HEK293T (DSMZ) and ATC cell line 8305 C (Fuheng Biotechnology) were grown in DMEM with 10% FBS under the same conditions.

### Plasmid and small interfering RNA (siRNA) transfection

SiRNAs targeting USP14, UCHL5, and PKCα (Sangon Biotech) were used, with sequences in Table S1. Overexpression plasmids GFP-PKCα, Flag-USP14 (Generay Biotech), and His-UCHL5 (Horizon Biotechnology) were synthesized. RNAi-resistant overexpression plasmids Flag-rUSP14, and His-rUCHL5 (Generay Biotech) were synthesized. Plasmids HA-UB and HA-K6, HA-K11, HA-K27, HA-K29, HA-K33, HA-K48, HA-K63 (Addgene) were obtained. Cells were grown in six-well plates to 30-40% density and transfected using jetPRIME (Polyplus, France) with 2 μg of siRNAs or plasmids in serum-free DMEM.

### Western blotting

Cells were lysed with protein lysis buffer, centrifuged at 4 °C, and supernatant protein concentrations were quantified using Coomassie Brilliant Blue. Proteins were separated by 10% SDS-PAGE, transferred to PVDF membranes (Millipore), and blocked with 5% skim milk for 1 hour. Membranes were incubated overnight at 4 °C with primary antibodies, washed with TBST, and probed with HRP-conjugated secondary antibodies for 90 minutes. Proteins were detected using ECL and analyzed with Image Lab software. Antibodies used are listed in Table S2.

### Tissue samples and immunohistochemistry (IHC)

Paraffin-fixed tissue sections were deparaffinized and rehydrated using xylene and alcohol. Antigens were retrieved with 1 mM EDTA (pH 8.0), and endogenous peroxidases were blocked with 0.3% hydrogen peroxide. Sections were incubated with 3% goat serum for 30 minutes, followed by primary antibodies overnight at 4 °C, and then secondary antibodies for 50 minutes at room temperature.

### Co-Immunoprecipitation (Co-IP)

At 4 °C, protein lysates were immunoprecipitated using specific antibodies against USP14, UCHL5, PKCα, or an IgG negative control. The lysates were mixed with 30 μL of pre-washed Protein A/G magnetic beads in RIPA buffer and incubated for 2 h at 4 °C on a rotator. Magnetic beads were collected and washed with RIPA, and the complexes that had been immunoprecipitated were extracted from the beads.

### Immunofluorescence staining

Cells were fixed with paraformaldehyde, blocked with BSA, and permeabilized with 0.2% Triton-X 100. Primary antibody for p65 was applied overnight at 4 °C, followed by a secondary antibody for 2 hours in the dark. After PBS washing, cells were stained with DAPI for 5 minutes and imaged using a fluorescence microscope.

### Animal model

Four-week-old female BALB/c nude mice were divided into two groups of six. Each mouse received a subcutaneous injection of 50 μL CAL62 cell suspension (12 × 10^6 cells in 1000 μL serum-free DMEM with 10% Matrigel). Once tumors reached a volume of 100 mm³, b-AP15 treatment (7.5 mg/kg) was given daily. Tumor volume, calculated as (Length × Width²) / 2, and body weight were monitored every 2-3 days. After 2 weeks, mice were euthanized, and tumors were excised, weighed, and photographed.

The animal experiment protocol was approved by the Institutional Animal Care and Use Committee at Zhejiang Provincial People’s Hospital, with the specific approval number 20240102173407450070.

### Clinical Specimens

The tissue microarray utilized in this study, consisting of 11 samples of normal thyroid tissue, 8 specimens of PTC, and 5 samples of ATC, was supplied by Zhejiang Provincial People’s Hospital. The research adhered to the guidelines set forth in the Declaration of Helsinki and received approval from the Institutional Ethics Committee at Zhejiang Provincial People’s Hospital.

### Data processing and statistical analysis

Statistical analysis was performed using unpaired Student’s t-tests in GraphPad Prism 10. Significance levels were denoted as: * for P < 0.05, ** for P < 0.01, and *** for P < 0.001. The graphical abstract was created using Figdraw software.

## Results

### USP14 and UCHL5 are highly expressed in ATC and associated with poor prognosis

**Fig. 1:**
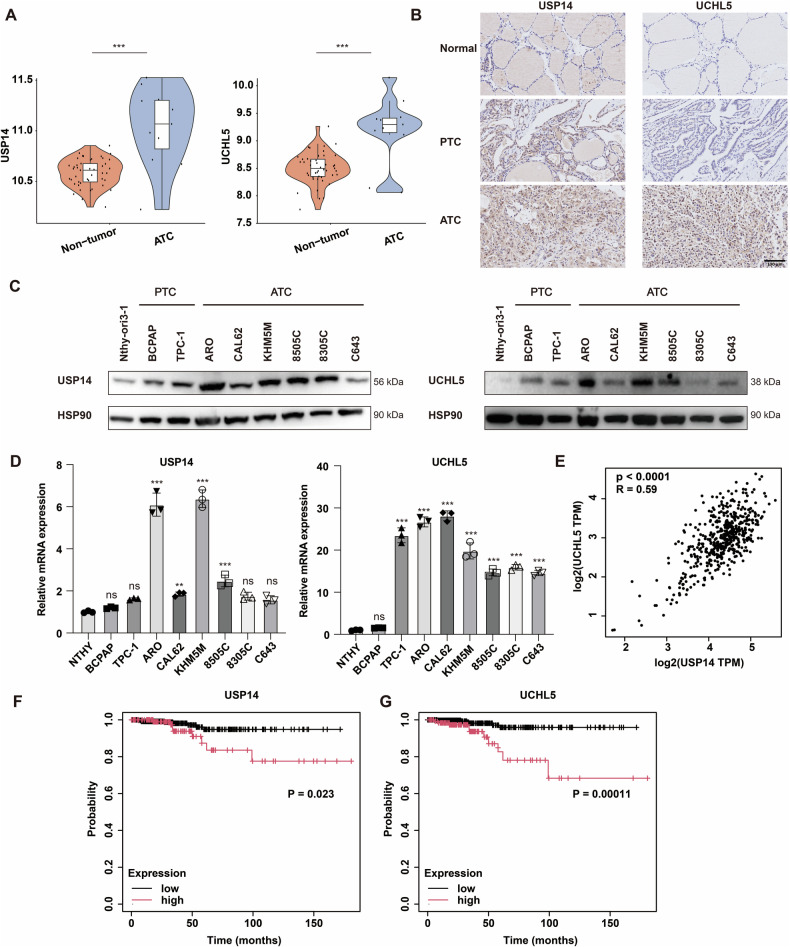
USP14 and UCHL5 expression in ATC and their clinical relevance. **A** Expression levels of USP14 and UCHL5 in healthy thyroid and ATC tissues using the GEO database (GSE33630). **B** IHC staining to evaluate the expression of USP14 and UCHL5 in normal thyroid, PTC, and ATC tissues. Scale bar, 100 μm. **C** Western blotting was used to detect USP14 and UCHL5 expression in one normal thyroid cell line (Nthy-ori3-1), two PTC cell lines (TPC-1, BCPAP), and six ATC cell lines (ARO, CAL62, KHM5M, 8505 C, 8305 C, and C643). **D** The qRT-PCR was used to detect the mRNA expression of USP14 and UCHL5 in normal thyroid, PTC and ATC cell lines. The experiment was repeated three times. **E** Correlation of USP14 and UCHL5 expression was analyzed by GEPIA2. **F**, **G** Kaplan-Meier plotter survival analysis illustrating the impact of USP14 and UCHL5 expression levels on the survival rates of thyroid cancer patients.

To investigate the expression profiles of USP14 and UCHL5 in ATC, we initially analyzed their expression levels in normal thyroid tissue and ATC using the GSE33630 dataset from the GEO database. Our analysis revealed that both USP14 and UCHL5 were significantly upregulated in ATC (Fig. 1A). Furthermore, we constructed a tissue microarray using 11 normal thyroid tissues, 8 PTC, and 5 ATC samples, and performed IHC staining on the microarray. The results indicated that USP14 exhibited an increased positive staining ratio, rising from 0% (0/11) in normal thyroid tissues to 87.5% (7/8) in PTC and 100% (5/5) in ATC samples. Similarly, UCHL5 demonstrated a significant increase in positive staining, from 0% (0/11) in normal tissues to 75% (6/8) in PTC and 100% (5/5) in ATC samples, highlighting the notable upregulation of USP14 and UCHL5 in thyroid cancer compared to normal thyroid tissues (Table S5). The representative IHC analysis confirmed that the expression levels of USP14 and UCHL5 were markedly elevated in ATC tissues compared to normal thyroid tissues and PTC tissues (Fig. 1B).

Next, we evaluated the protein and mRNA expression levels of USP14 and UCHL5 across various cell lines, including the normal thyroid cell line Nthy-ori3-1, PTC cell lines TPC-1 and BCPAP, and ATC cell lines CAL62, KHM5M, 8505 C, 8305 C, and C643. The results indicated that the expression of USP14 and UCHL5 was upregulated in ATC cell lines (Fig. 1C, D). To explore the potential correlation between USP14 and UCHL5, we conducted a correlation analysis using the GEPIA2 database [24], which revealed a significant positive correlation between the expression levels of USP14 and UCHL5 (Fig. 1E). Finally, we performed a prognostic analysis using data from the Kaplan-Meier plotter [25]. The analysis showed that patients with high expression levels of USP14 and UCHL5 had poorer clinical outcomes and lower survival rates (Fig. 1F, G).

### Silencing of USP14 and UCHL5 individually or simultaneously inhibits the malignant phenotype of ATC

**Fig. 2:**
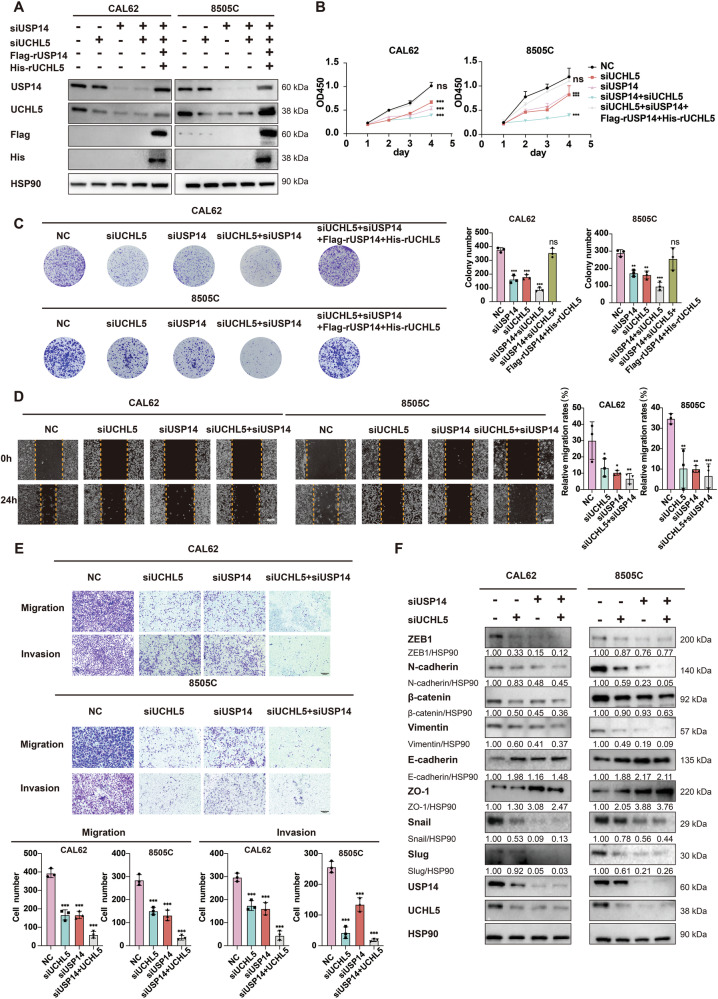
Silencing USP14 and UCHL5 decreases the malignant properties of ATC cells. **A** The siRNAs targeting USP14 and UCHL5, alongside RNAi-resistant overexpression constructs of USP14 and UCHL5 plasmids, were transfected into two ATC cell lines, 8505 C and CAL62. Western blotting confirmed the expression of USP14 and UCHL5. **B**, **C** Cell viability was assessed using CCK-8 and colony formation assays. The experiment was repeated three times. **D** Wound healing assay showing the effect of USP14 and UCHL5 knockdown on the migration of CAL62 and 8505 C cells at 0 and 24 h. Scale bar, 400 μm. The experiment was repeated three times. **E** Transwell assays showing the effect of USP14 and UCHL5 knockdown on the migration and invasion of CAL62 and 8505 C cells. Scale bar, 400 μm. The experiment was repeated three times. **F** Western blotting was used to detect changes in EMT-related markers in 8505 C and CAL62 cells transfected with USP14 and UCHL5 siRNAs.

To investigate the functions of USP14 and UCHL5 in ATC, we used siRNAs to knock down these two genes and validated the knockdown efficiency using western blotting (Fig. 2A). After successful knockdown of USP14 and UCHL5, CCK-8 and colony formation assays confirmed that the knockdown of USP14 or UCHL5 inhibited ATC cell proliferation. Notably, simultaneous knockdown of both USP14 and UCHL5 resulted in a more pronounced inhibition of cell proliferation compared to individual knockdowns (Fig. 2B, C). Meanwhile, the introduction of RNAi-resistant overexpression consctruct plasmids (Flag-rUSP14 and His-rUCHL5) successfully rescued cell proliferation (Fig. 2A–C). It helped to ensure that the observed effects on cell viability were not due to off-target effects of siRNA.

Additionally, wound healing assays showed that individual or combined knockdown of USP14 and UCHL5 suppressed ATC cell migration, with the combined knockdown showing a stronger effect (Fig. 2D). Transwell assays further showed that silencing of the two genes results in a reduction in the number of cells that migrated through the membrane, indicating a decrease in the migration and invasion capabilities of ATC cells. The combined knockdown had a more substantial impact (Fig. 2E). Western blotting also revealed the expression change of epithelial-mesenchymal transition (EMT) markers, including ZEB-1, β-catenin, N-cadherin, Vimentin, Snail, and Slug with the combined knockdown leading to a more pronounced downregulation, while the expression of E-cadherin and ZO-1 was remarkably upregulated (Fig. 2F).

### b-AP15 inhibits malignant phenotypes of ATC

**Fig. 3:**
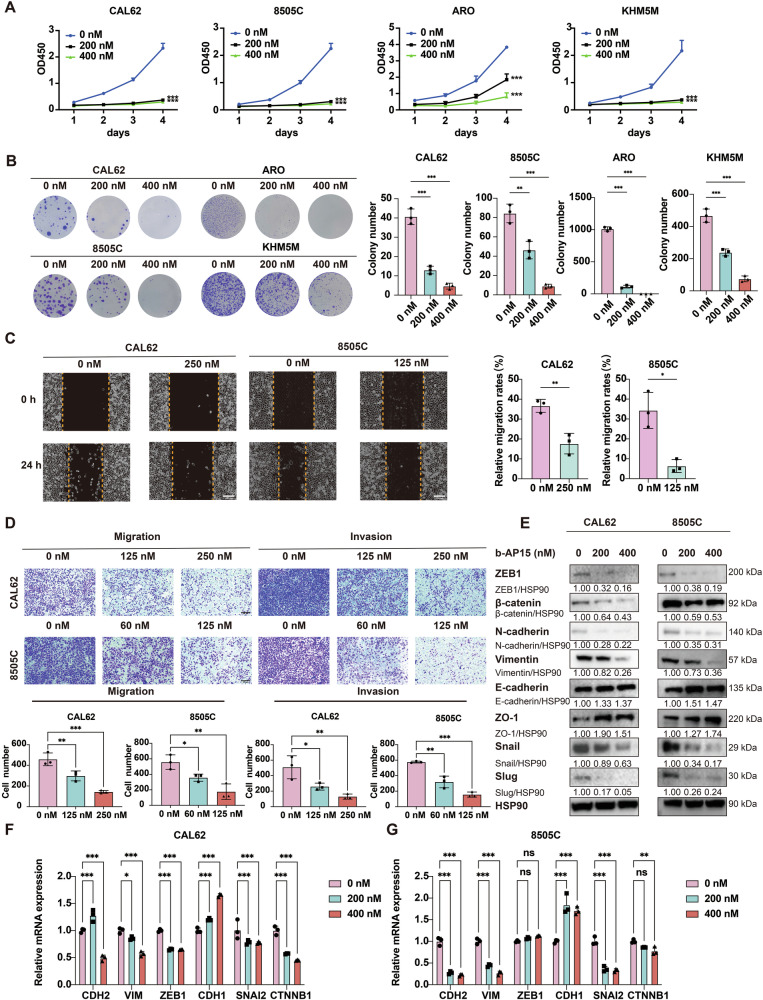
b-AP15 suppressed the malignant properties of ATC cells. **A, B** Using CCK-8 and colony formation assays, cell proliferation of CAL62, 8505 C, ARO, and KHM5M cells treated with of b-AP15 (0 nM, 200 nM, and 400 nM) was assessed over a period of 4 days. The experiment was repeated three times. **C** Wound healing assay showing the effect of b-AP15 on the migration of CAL62 and 8505 C cells at 0 and 24 h. Scale bar, 400 μm. The experiment was repeated three times. **D** Transwell assays showing the effect of b-AP15 on the migration and invasion of CAL62 and 8505 C cells. Scale bar, 400 μm. The experiment was repeated three times. **E** Western blotting was used to detect the expression of EMT-related markers in CAL62 and 8505 C cells treated with b-AP15. **F, G** The qRT-PCR was used to detect the mRNA of EMT-related genes in CAL62 and 8505 C cells treated with b-AP15. The experiment was repeated three times.

b-AP15 is a potent dual inhibitor of USP14 and UCHL5 [13]. We determined the IC50 values of b-AP15 in four ATC cell lines (8505 C, CAL62, KHM5M, and ARO) and found that nanomolar concentrations effectively inhibited ATC cell proliferation (Fig. S1A–D). CCK-8 and colony formation assays further confirmed that b-AP15 significantly suppressed ATC cell proliferation (Fig. 3A, B). Additionally, wound healing and transwell assays demonstrated that b-AP15 treatment effectively inhibited the migration and invasion capabilities of ATC cells (Fig. 3C, D). Western blotting analysis revealed that treatment with b-AP15 resulted in the downregulation of ZEB-1, β-catenin, N-cadherin, vimentin, Snail, and Slug, along with the upregulation of E-cadherin and ZO-1 (Fig. 3E). The qRT-PCR analysis showed that b-AP15 treatment increased the expression of CDH1 gene and decreased the expression of CDH2, VIM, SNAI2, and CTNNB1 genes (Fig. 3F, G).

Moreover, flow cytometry analysis indicated that b-AP15 treatment promoted apoptosis of ATC cells (Fig. S2A, B). Meanwhile, western blotting analysis also revealed that b-AP15 dose-dependently induced the makers of apoptosis, cleaved PARP (c-PARP) and cleaved caspase 3 (c-caspase 3) (Fig. S2C). Furthermore, b-AP15 induced G2/M phase cell cycle arrest in ATC cells (Fig. S2D, E) .The qRT-PCR analysis showed that b-AP15 treatment reduced the gene expression levels of cell cycle-related proteins CDC25C, CDK4, and CCNB1 (Fig. S2F).

### USP14 and UCHL5 collaboratively regulate PKCα

**Fig. 4:**
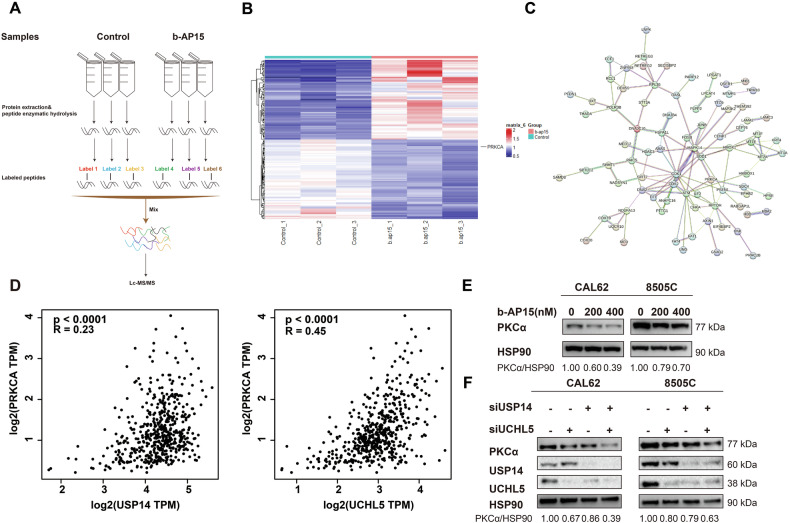
USP14 and UCHL5 cooperatively downregulate PKCα. **A** The TMT proteomics analysis was performed in CAL62 cells treated with b-AP15. **B** Heatmap showing the expression levels of differentially expressed proteins in control and b-AP15-treated CAL62 cells. **C** PPI analysis of the top 150 differentially expressed genes based on the STRING database. **D** Correlation analysis of USP14/UCHL5, and PKCα using the GEPIA2 database. **E** Western blotting was used to detect the expression of PKCα in CAL62 and 8505 C cells treated with b-AP15 at different concentrations. **F** Western blotting was used to detect the expression of PKCα in CAL62 and 8505 C cells with USP14 and UCHL5 knockdown.

To further investigate the mechanisms by which USP14 and UCHL5 influence the malignant phenotype of ATC cells, we performed TMT proteomics analysis on CAL62 cells treated with b-AP15 (Fig. 4A and Table S4). Heatmap analysis showed that PKCα was significantly downregulated in CAL62 cells treated with b-AP15 (Fig. 4B). Using the STRING database [26], we performed protein-protein interaction (PPI) analysis of the differentially expressed proteins, which indicated that PKCα was central to the interaction network, interacting with multiple important proteins (Fig. 4C). Therefore, We selected PKCα as the target protein for USP14 and UCHL5 due to its prominence in the PPI network and the lack of prior research connecting USP14 to the PKC family, offering new insights into tumor biology mechanisms.

Next, we analyzed the correlation between USP14, UCHL5, and PKCα using the GEPIA2 database [24]. The results showed a positive correlation between USP14 and PKCα, as well as UCHL5 and PKCα (Fig. 4D). To verify the accuracy of the proteomics results, we treated 8505 C and CAL62 cells with b-AP15 and performed knockdown of USP14 and UCHL5. Western blotting confirmed that PKCα expression was downregulated in both treatments (Fig. 4E, F). Notably, simultaneous knockdown of both USP14 and UCHL5 resulted in a more significant downregulation of PKCα expression compared to the individual knockdowns (Fig. 4F).

### USP14 and UCHL5 interact with PKCα and synergistically enhance its stability

**Fig. 5:**
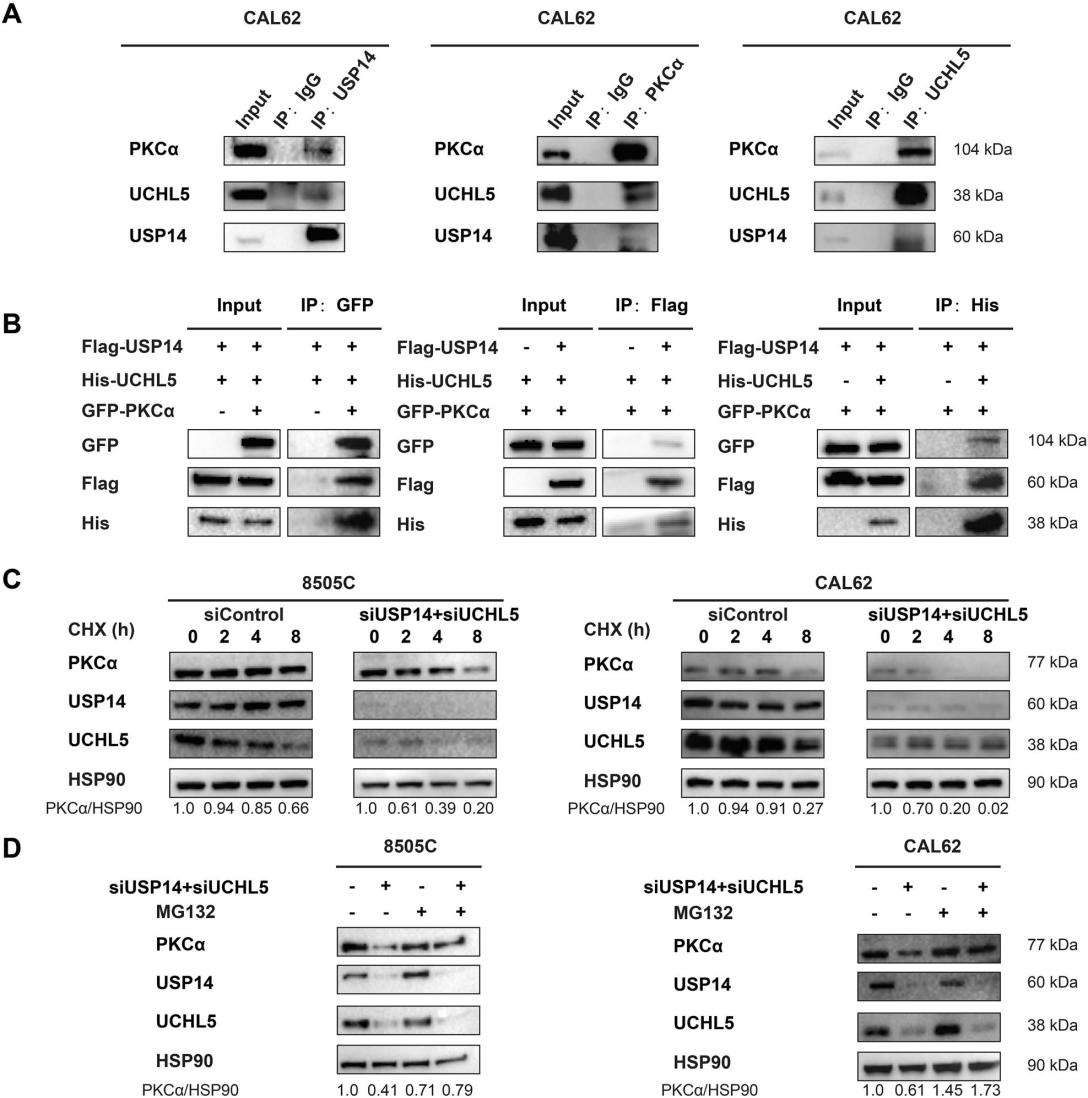
USP14 and UCHL5 interact with PKCα and stabilize it. **A** Co-IP assays were detected to the interactions between endogenous USP14, UCHL5, and PKCα in CAL62 cells using specific antibodies. **B** Co-IP assays were detected to the interactions between exogenous USP14, UCHL5, and PKCα in HEK293T cells co-transfected plasmids expressing Flag-USP14, His-UCHL5, and GFP-PKCα using antibodies against Flag, His, and GFP. **C** Western blotting was used to detect half-life of PKCα in 8505 C and CAL62 cells treated with 100 µM cycloheximide (CHX) for specified time periods. **D** Western blotting was used to detect the expression of PKCα in 8505 C and CAL62 cells treated with 10 µM MG132 for 6 hours.

Given the deubiquitinating activities of USP14 and UCHL5, we hypothesized that USP14 and UCHL5 may deubiquitinate and stabilize PKCα. To test this hypothesis, we first performed co-IP assays in CAL62 cells to verify whether endogenous USP14 or UCHL5 interact with PKCα. The results showed that USP14 interacts with UCHL5 and PKCα, UCHL5 interacts with USP14 and PKCα, and PKCα interacts with USP14 and UCHL5, indicating endogenous mutual interactions among these three proteins (Fig. 5A). Next, we obtained plasmids expressing Flag-USP14, His-UCHL5, and GFP-PKCα and co-transfected them into HEK293T cells. Using antibodies against GFP, Flag, and His antibody, co-IP assays confirmed these exogenous interactions: USP14 interacts with UCHL5 and PKCα, UCHL5 interacts with USP14 and PKCα, and PKCα interacts with USP14 and UCHL5 (Fig. 5B). To further investigate the stability of PKCα, we treated 8505 C and CAL62 cells with cycloheximide (CHX), a protein synthesis inhibitor. CHX treatment accelerated the degradation of PKCα following knockdown of USP14 and UCHL5. However, treatment with MG132, a proteasome inhibitor, prevented the degradation of PKCα caused by the knockdown of USP14 and UCHL5 (Fig. 5C, D). These finding indicated that USP14 and UCHL5 synergistically stabilize PKCα by reducing its proteasomal degradation pathway.

### USP14 and UCHL5 can remove K48-Linked ubiquitin chains from PKCα

**Fig. 6:**
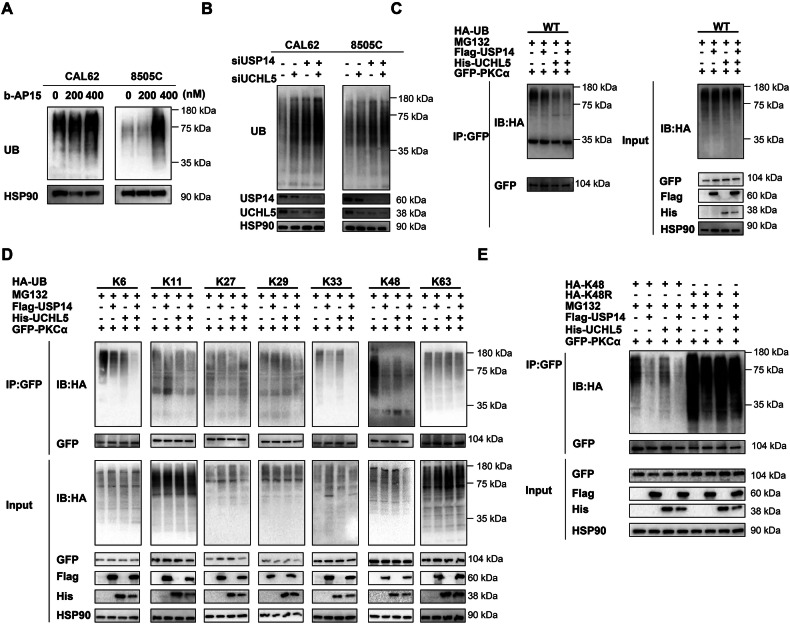
USP14 and UCHL5 deubiquitinate PKCα. **A** Western blotting was used to detect UB levels in 8505 C and CAL62 cells treated with b-AP15. **B** Western blotting was used to detect UB levels in 8505 C and CAL62 cells transfected with siRNAs of USP14 and UCHL5. **C, D** HEK293T cells were co-transfected with HA-WT, K6, K11, K27, K29, K33, K48, and K63 UB plasmids, along with Flag-USP14, His-UCHL5, and GFP-PKCα plasmids. Cells were treated with 10 μM MG132 for 6 hours. UB levels of PKCα were detected using anti-HA antibody. **E**. HEK293T cells were co-transfected with HA-K48 UB, HA-K48R UB mutant, Flag-USP14, His-UCHL5, and GFP-PKCα plasmids. Cells were treated with 10 μM MG132 for 6 h. UB levels of PKCα were detected using anti-HA antibody.

To validate how USP14 and UCHL5 regulate PKCα, we first determined whether these DUBs could affect overall cellular ubiquitin levels. In 8505 C and CAL62 cells, we observed that both treatment with b-AP15 and the knockdown of USP14 and UCHL5 led to an increase in overall cellular ubiquitin levels (Fig. 6A, B). Next, we treated HEK293T cells with MG132 and co-transfected HA-UB, Flag-USP14, His-UCHL5, and GFP-PKCα plasmids. The results showed that USP14 and UCHL5 could suppress the ubiquitination level of PKCα, and they exhibit a synergistic effect (Fig. 6C). To investigate the specific ubiquitination sites, we used UB plasmids containing K6, K11, K27, K29, K33, K48, and K63 ubiquitin variants. HEK293T cells were treated with MG132 and co-transfected with these UB plasmids, along with Flag-USP14, His-UCHL5, and GFP-PKCα plasmids. The results indicated that USP14 and UCHL5 could suppress the ubiquitination level of PKCα at K6, K33, and K48 sites, with the most significant reduction observed at the K48 site, which is associated with protein degradation (Fig. 6D). Thus, to further confirm the role of K48 ubiquitination, we treated HEK293T cells with MG132 and co-transfected them with HA-K48 ubiquitin, HA-K48R mutant ubiquitin, Flag-USP14, His-UCHL5, and GFP-PKCα plasmids. The results showed that USP14 and UCHL5 can suppress the ubiquitination level of PKCα at K48 site, while the K48R mutant showed no significant changes (Fig. 6E). Therefore, our findings suggested that USP14 and UCHL5 can maintain PKCα stability by removing K48-linked ubiquitin chains from PKCα.

### USP14 and UCHL5 regulates NF-κB signaling pathway through stabilizing PKCα

**Fig. 7:**
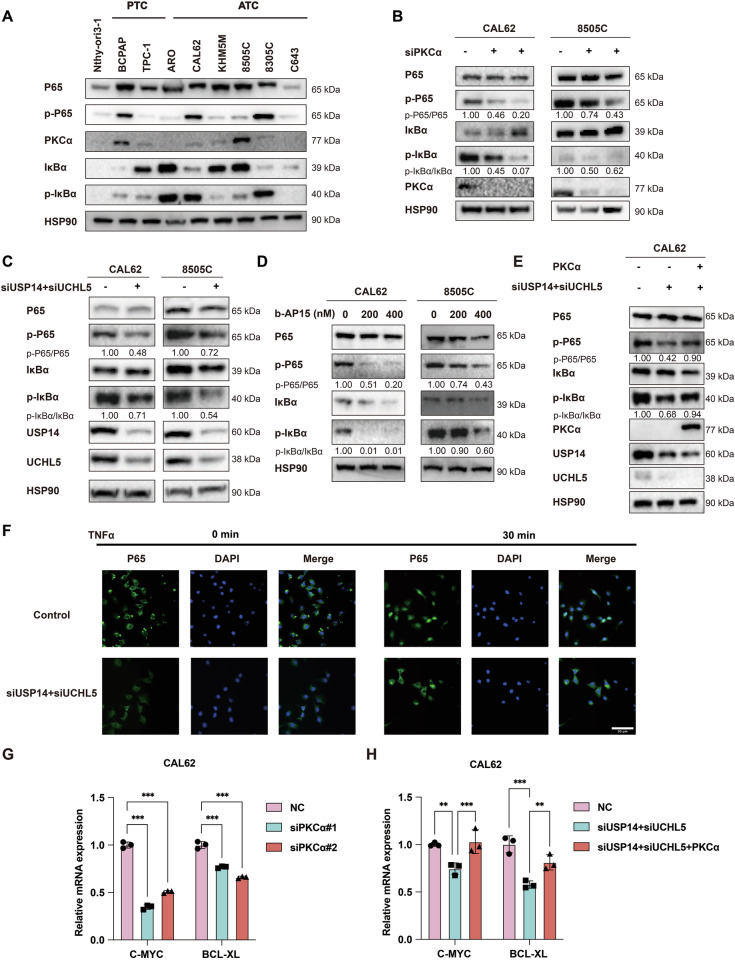
USP14 and UCHL5 regulate the NF-κB signaling pathway by stabilizing PKCα. **A** Western blotting was used to detect the expression levels of P65, p-P65, IκBα, and p-IκBα in various cells. **B** Western blotting was used to detect the protein levels of P65, p-P65, IκBα, and p-IκBα in CAL62 and 8505 C cells transfected with the siRNAs of PKCα. **C** Western blotting was used to detect the protein levels of P65, p-P65, IκBα, and p-IκBα in CAL62 and 8505 C cells transfected with the siRNAs of USP14 and UCHL5. **D** Western blotting was used to detect the protein levels of P65, p-P65, IκBα, and p-IκBα in CAL62 and 8505 C cells treated with b-AP15. **E** Western blotting was used to detect the protein levels of P65, p-P65, IκBα, and p-IκBα in CAL62 cells co-transfected with USP14 siRNAs, UCHL5 siRNAs, and PKCα plasmids. **F** Immunofluorescence staining was used to detect the nuclear translocation of P65 in CAL62 cells transfected with the siRNAs of USP14 and UCHL5 and then stimulated with TNFα. Scale bar, 50 μm. **G** The qRT-PCR was used to detect the mRNA levels of P65 downstream genes, such as C-MYC and BCL-XL, in CAL62 cells transfected with the siRNAs of PKCα. The experiment was repeated three times. **H** The qRT-PCR was used to detect the mRNA levels of C-MYC and BCL-XL in CAL62 cells co-transfected with USP14 siRNAs, UCHL5 siRNAs, and PKCα plasmids. The experiment was repeated three times.

Previous studies have demonstrated that PKCα can regulate the NF-κB signaling pathway in inflammatory skin disorders [27]. In this study, we explored whether a similar regulatory relationship exists in ATC and how it might be modulated. Therefore, we aimed to determine whether USP14 and UCHL5 promote ATC development by regulating NF-κB via PKCα. First, we examined the protein expression levels of P65, phospho-P65 (p-P65), IκBα, phospho-IκBα (p-IκBα), and PKCα in normal thyroid, PTC and ATC cells. The results showed that PKCα was upregulated and that NF-κB signaling pathway was activated in ATC cell lines (Fig. 7A). Next, we knocked down PKCα in CAL62 and 8505 C cells and found that the expression levels of p-IκBα and p-P65 were downregulated (Fig. 7B). Then, we knocked down USP14 and UCHL5 or treated the cells with b-AP15 in CAL62 and 8505 C cells. The results indicated that the expression levels of p-IκBα and p-P65 were also downregulated (Fig. 7C, D). We transfected USP14/UCHL5 siRNA and PKCα plasmids into CAL62 cells. We observed that PKCα could rescue the downregulation of p-IκBα and p-P65 caused by USP14 and UCHL5 knockdown (Fig. 7E). Similarly, immunofluorescence staining showed that in TNFα-stimulated CAL62 cells (TNFα is an activator of NF-κB signaling pathway), knockdown of USP14/UCHL5 reduced the nuclear translocation of P65 compared to the control group (Fig. 7F). Finally, we performed qRT-PCR to detect the expression of downstream gene of P65, specifically C-MYC and BCL-XL. The results showed that the expression of C-MYC and BCL-XL were reduced when PKCα was knocked down (Fig. 7G) or when USP14 and UCHL5 were knocked down (Fig. 7H). Notably, PKCα overexpression could rescue the downregulation of C-MYC and BCL-XL caused by the knockdown of USP14 and UCHL5 (Fig. 7H). In summary, USP14 and UCHL5 regulate the NF-κB signaling pathway by stabilizing PKCα, thereby modulating the pro-oncogenic and anti-apoptotic signals of NF-κB.

### USP14 and UCHL5 promote the malignant characteristics of ATC cells by PKCα

**Fig. 8:**
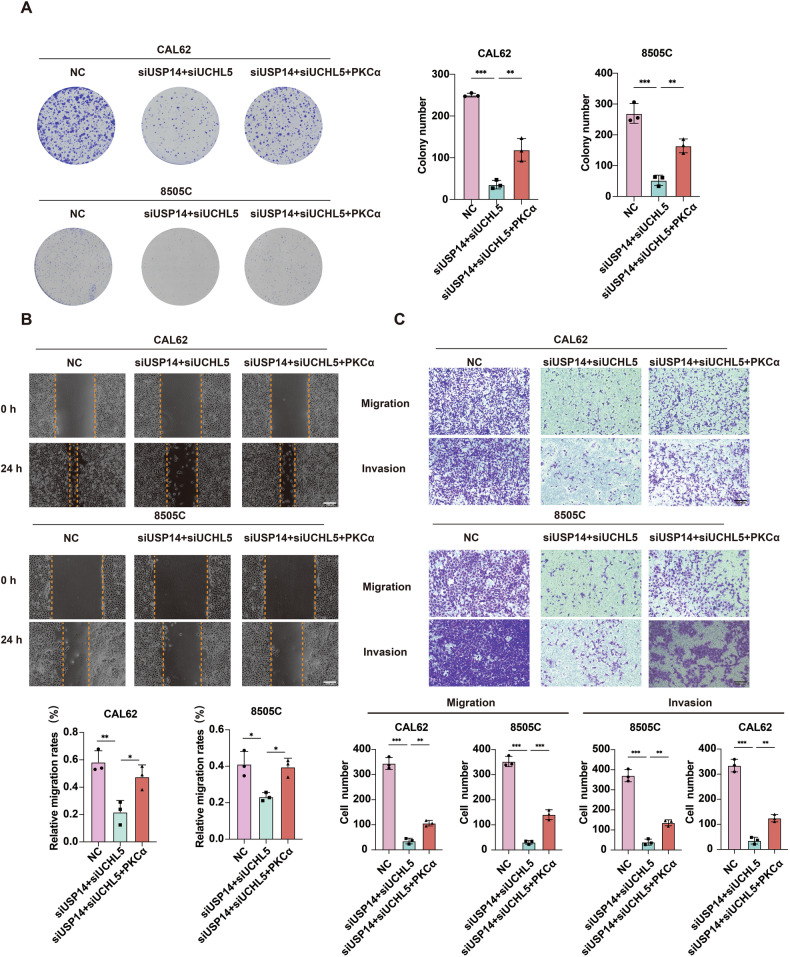
USP14 and UCHL5 promote the malignant phenotype of ATC cells by PKCα. **A** Colony formation assays were performed to assess the proliferation of cells co-transfected with siRNAs targeting USP14 and UCHL5, alongside PKCα plasmids. The experiment was repeated three times. **B** Wound healing assays illustrating the migration capability of CAL62 and 8505 C cells co-transfected with siRNAs targeting USP14 and UCHL5, as well as PKCα plasmids, at 0 and 24 h. Scale bar, 400 μm. The experiment was repeated three times. **C** Transwell assays demonstrating the impact of co-transfection with siRNAs targeting USP14 and UCHL5, along with PKCα plasmids, on the migration and invasion capability of CAL62 and 8505 C cells. Scale bar, 400 μm. The experiment was repeated three times.

To investigate whether USP14 and UCHL5 mediate malignant characteristics under the influence of PKCα, we performed knockdown experiments for USP14 and UCHL5 alongside PKCα overexpression rescue assays. Our results demonstrated that the transfection of siRNAs targeting USP14 and UCHL5 significantly inhibited cell proliferation (Fig. 8A). Importantly, this inhibitory effect was substantially reversed upon additional transfection with a PKCα expression plasmid (Fig. 8A). It indicated that PKCα can effectively compensate for the reduced expression of USP14 and UCHL5, thereby restoring cell proliferation capabilities. The wound healing and transwell assays revealed that the migration and invasion capabilities of cells, which were significantly reduced by the knockdown of USP14 and UCHL5, were markedly restored upon co-transfection with siRNAs targeting USP14 and UCHL5 along with the PKCα plasmid (Fig. 8B, C). This finding further confirmed that PKCα overexpression can effectively rescue the diminished migration and invasion abilities caused by the knockdown of USP14 and UCHL5. Similarly, consistent experimental results were observed in the presence of b-AP15 alongside PKCα overexpression. Treatment with b-AP15 led to a significant reduction in cell proliferation, migration, and invasion capabilities (Fig. S3A–C). Importantly, when cells were treated with b-AP15 and simultaneously transfected with the PKCα plasmid, the diminished cellular functions were effectively restored (Fig. S3A–C). These findings collectively suggested that PKCα can counteract the inhibitory effects imposed by the blockade or depletion of USP14 and UCHL5, thereby maintaining cellular functionalities.

### b-AP15 inhibits tumor growth of ATC in vivo

**Fig. 9:**
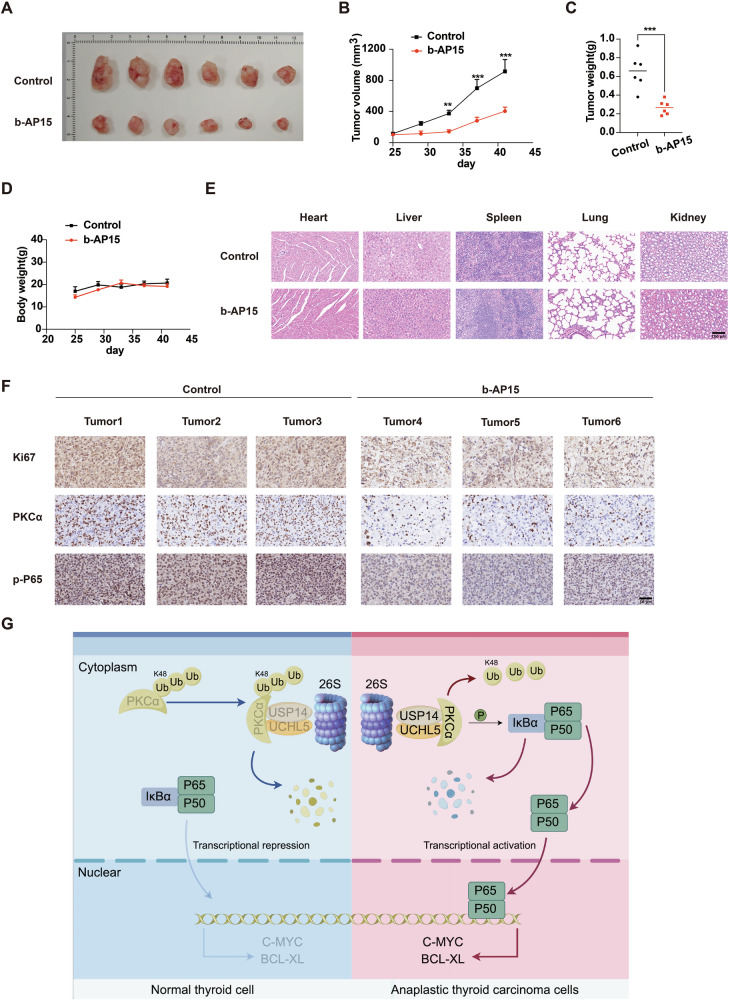
Effects of b-AP15 on tumor growth in vivo. **A** The images of tumors from nude mice treated with vehicle and b-AP15. **B** Time-dependent changes in tumor volume in nude mice treated with vehicle and b-AP15. (*n* = 6). **C** Quantification of tumor weight in nude mice treated with vehicle and b-AP15. (*n* = 6). **D** Body weight of nude mice over time in the control and b-AP15 groups. (*n* = 6) **E** H&E staining of heart, liver, spleen, lung, and kidney tissues from nude mice treated with vehicle and b-AP15. Scale bar, 100 μm. **F**. IHC staining of Ki67, PKCα, and p-P65 in tumor tissues from nude mice treated with vehicle and b-AP15. Scale bar, 50 μm. **G** Schematic representation of the USP14 and UCHL5 roles in normal thyroid cells and ATC cells. In normal thyroid cell, PKCα is ubiquitinated and degraded via the 26S proteasome, leading to transcriptional repression of C-MYC and BCL-XL. In ATC cell, PKCα is stabilized by USP14 and UCHL5, leading to the activation of NF-κB and subsequent transcriptional activation of C-MYC and BCL-XL.

In vivo studies employing a xenograft mouse model illustrated that the administration of b-AP15 significantly inhibited tumor growth, leading to reductions in both tumor volume and weight in nude mice (Fig. 9A–C), without impacting their body weight (Fig. 9D). Histological examination using H&E staining of heart, liver, spleen, lung, and kidney tissues from these nude mice did not reveal any notable toxic side effects caused by b-AP15 treatment (Fig. 9E). Consistent with the cellular results, IHC staining demonstrated that b-AP15 treatment markedly diminished the expression of Ki67, a marker closely related to cell proliferation (Fig. 9F). Importantly, both IHC staining and immunofluorescence experiments revealed downregulation of PKCα and p-P65 in tumors from b-AP15-treated nude mice (Fig. 9F and Fig. S4). These findings supported the conclusion that USP14/UCHL5 facilitate the progression of ATC through the PKCα-P65 pathway.

## Discussion

Anaplastic thyroid cancer (ATC) is an exceptionally aggressive and rare form of thyroid cancer, distinguished by its rapid growth and early metastasis [5]. The median survival time for patients with ATC is less than one year [5]. The molecular pathogenesis underlying ATC involves a complex interplay of gene mutations, including those in TP53, RB1, RAS, BRAF, as well as mutations in the TERT promoter region [28–30]. These mutations collectively disrupt normal cell proliferation controls. Despite significant progress in understanding the mechanisms of thyroid cancer, effective therapeutic options for ATC remain limited [4]. In current study, we explored the role of two DUBs, USP14 and UCHL5, in the pathogenesis of ATC. Our findings demonstrate that USP14 and UCHL5 are upregulated in ATC at both the protein and mRNA levels, suggesting their potential involvement in the aggressive behavior of ATC.

The knockdown of USP14 and UCHL5, or treatment with the inhibitor b-AP15, significantly impeded ATC cell proliferation, reduced metastasis capabilities, and suppressed tumor formation in nude mice models. Mechanistically, our findings offer compelling evidence that the dual inhibition of USP14 and UCHL5 through b-AP15 represents a promising therapeutic strategy for combating ATC. The mechanistic insights gained from our study reveal a critical role for these DUBs in the stabilization of PKCα, which in turn drives the activation of NF-κB signaling. In summary, in normal thyroid cells, PKCα undergoes ubiquitination and degradation via the 26S proteasome, leading to the transcriptional repression of pro-oncogenic and anti-apoptotic genes regulated by p65, such as C-MYC and BCL-XL. Conversely, in ATC cells, the overexpression of USP14 and UCHL5 prevents PKCα degradation by collaboratively removing K48-linked ubiquitin chains. This stabilization of PKCα promotes the nuclear translocation of p65, resulting in the upregulation of C-MYC and BCL-XL, key factors in driving cell proliferation and survival (Fig. 8G). b-AP15 is a dual-targeting inhibitor that specifically acts upon USP14 and UCHL5. By effectively disrupting these targeted pathways, b-AP15 leads to a significant suppression in both tumor growth and metastasis. These results highlight the potential of targeting the USP14/UCHL5-PKCα-NF-κB axis as a novel therapeutic avenue for ATC, offering a new prospect for the development of innovative treatment strategies against highly lethal ATC.

The roles of USP14 and UCHL5 in cancer have indeed been examined in various contexts, including colorectal cancer and chronic myeloid leukemia [23, 31]. In these cancers, USP14 and UCHL5 have been shown to stabilize crucial oncogenic proteins and promote tumor progression. Our findings in ATC are consistent with these observations, suggesting that the dysregulation of DUBs may be a common characteristic in various aggressive cancers. However, the particular downstream targets and signaling pathways implicated can differ across various cancer types, emphasizing the significance of context-specific mechanisms in the field of cancer biology.

The identification of USP14 and UCHL5 as potential therapeutic targets in ATC opens new avenues for the development of targeted therapies. Current treatments for ATC, including surgery, radiotherapy, and chemotherapy, show limited efficacy due to the aggressive characteristic of the disease [4]. Targeting USP14 and UCHL5 with specific inhibitors, such as b-AP15, represents a potentially more effective strategy for addressing the underlying molecular mechanisms that drive the progression of ATC. Future studies should concentrate on optimizing the specificity and efficacy of USP14/UCHL5 inhibitors and evaluating their safety and effectiveness in both preclinical models and clinical trials.

While our study offers valuable insights into the role of USP14/UCHL5 in ATC, it is important to acknowledge several limitations. First, the in vitro and in vivo models utilized in this study may not fully recapitulate the complexity of the human disease. Second, the clinical relevance of USP14/UCHL5 upregulation in ATC necessitates further validation through studies involving larger patient cohorts. Additionally, it is essential to carefully evaluate the potential off-target effects of USP14/UCHL5 inhibitors and their impact on normal tissues. Future research should also investigate the potential of combining USP14/UCHL5 inhibitors with existing therapies to maximize therapeutic benefits.

In conclusion, our study underscores the significance of USP14 and UCHL5 in the pathogenesis of ATC, suggesting that targeting these DUBs could provide a novel therapeutic approach for this highly lethal disease. Further investigations are indeed warranted to translate these findings into clinical applications, with the goal of improving outcomes for patients with ATC.

## Supplementary information

SUPPLEMENTAL MATERIAL

Original WB data

## Data Availability

All data supporting the findings of this study are available from the corresponding author on reasonable request.
