# Supplementary material for: USP14 and UCHL5 synergistically deubiquitinate PKCα and translocate NF-κB to promote the progression of anaplastic thyroid cancer
Source: Cell Death Dis. 2025 Aug 13;16(1):617. doi: 10.1038/s41419-025-07890-9 (PMC12350928; doi:10.1038/s41419-025-07890-9)
Supplement: Supplementary file 1 — SUPPLEMENTAL MATERIAL [file 41419_2025_7890_MOESM1_ESM.docx]

**SUPPLEMENTAL MATERIAL: USP14 and UCHL5 Synergistically Deubiquitinate PKCα and Translocate NF-κB to Promote the Progression of Anaplastic Thyroid Cancer**

Song Zhang^1,2,3,4#^, Bing Han^1#^, Bo Jiang^1^, Mengyu Chen^1^, Deyang Mu^5^, Qi Wang^1^, Shu Sun^1^, Tong Xu^1^, Feifeng Song^1^, Xinxin Ren^1^, Zongfu Pan^1^, Ping Huang^1,2,6,^*, and Minghua Ge^2,6,7,^*

^1^ Center for Clinical Pharmacy, Cancer Center, Department of Pharmacy, Zhejiang Provincial People’s Hospital (Affiliated People’s Hospital), Hangzhou Medical College, Hangzhou, China.

^2^ Zhejiang Key Laboratory of Precision Medicine Research on Head & Neck Cancer, Zhejiang Provincial People’s Hospital, Hangzhou, China.

^3^ Cancer Center, Department of Pathology, Zhejiang Provincial People’s Hospital (Affiliated People’s Hospital), Hangzhou Medical College, Hangzhou, China.

^4^ Clinical Research Institute, Zhejiang Provincial People’s Hospital (Affiliated People’s Hospital), Hangzhou Medical College, Hangzhou, China.

^5^ College of Pharmaceutical Sciences, Hangzhou Medical College, Hangzhou, China.

^6^ Otolaryngology & Head and Neck Center, Cancer Center, Department of Head and Neck Surgery, Zhejiang Provincial People’s Hospital (Affiliated People’s Hospital), Hangzhou Medical College, Hangzhou, China.

^7^ Zhejiang Provincial Clinical Research Center for Head & Neck Cancer, Zhejiang Provincial People’s Hospital, Hangzhou, China.

^#^ These authors contributed equally to this work.

Running title: USP14 and UCHL5 Synergistically Deubiquitinate PKCα

* Corresponding Authors: Minghua Ge, Otolaryngology & Head and Neck Center, Cancer Center, Department of Head and Neck Surgery, Zhejiang Provincial People’s Hospital (Affiliated People’s Hospital), Hangzhou Medical College, Hangzhou, China. Email: geminghua@hmc.edu.cn. Ping Huang, Center for Clinical Pharmacy, Cancer Center, Department of Pharmacy, Zhejiang Provincial People’s Hospital (Affiliated People’s Hospital), Hangzhou Medical College, Hangzhou, China. Email: huangping@hmc.edu.cn.

#### Materials and Methods

**Wound healing assay**

8505C and CAL62 cells were treated with either siRNA transient transfection or b-AP15. After 24 hours, the cells were trypsinized, reseeded in 24-well plates to form a confluent monolayer, and scratched using a pipette tip. Debris was removed by washing with PBS, and images were captured immediately (0 hour). Cells were then cultured in serum-free medium for 24 hours, and the scratch condition was recorded again. The scratch width was measured using ImageJ software to calculate the relative migration rate.

**Transwell assay**

Transwell chambers (Corning, Catalog: 3422) were used for migration and invasion assays. For migration, the upper chamber was uncoated; for invasion, it was precoated with 250 μg/mL Matrigel (Corning, Catalog: 356234) and incubated at 37°C for 45 minutes. The upper chamber contained approximately 3 × 10^4 cells of 8505C or CAL62, while the lower chamber had medium with 10% FBS. After 48 hours, chambers were fixed with 4% paraformaldehyde and stained with 0.2% crystal violet.

**qRT-PCR**

RNA was extracted using the RNA Rapid Extraction Kit (Accurate Biotechnology) and converted to cDNA with the Reverse Transcription Kit (Accurate Biotechnology). qRT-PCR was done using Super SYBR Green Master Mix (Accurate Biotechnology) to quantify gene expression, calculated by the 2^(-ΔΔCt) method and normalized to GAPDH. Primer sequences are in Table S3.

**Cell viability and colony formation assay**

1000 cells were seeded in 96-well plates for viability testing. CCK-8 assays, conducted on days 1, 2, 3, and 4 after a 1-hour incubation at 37°C, involved mixing 10 μL CCK-8 with 90 μL serum-free DMEM, and measuring absorbance at 450 nm. For colony formation, 1000 cells were seeded in six-well plates, grown for 10 days, then fixed with 4% paraformaldehyde and stained with 0.2% crystal violet.

**Flow cytometry**

To assess apoptosis after two days of b-AP15 treatment, the Annexin-V/FITC kit (Multi Sciences Biotech) was used, and apoptotic cells were quantified by flow cytometry (FCM). For cell cycle analysis, the Cell Cycle Staining Kit (Multi Sciences Biotech) was used, with quantification by FCM. Data for apoptosis and cell cycle were analyzed using Flowjo software.

**
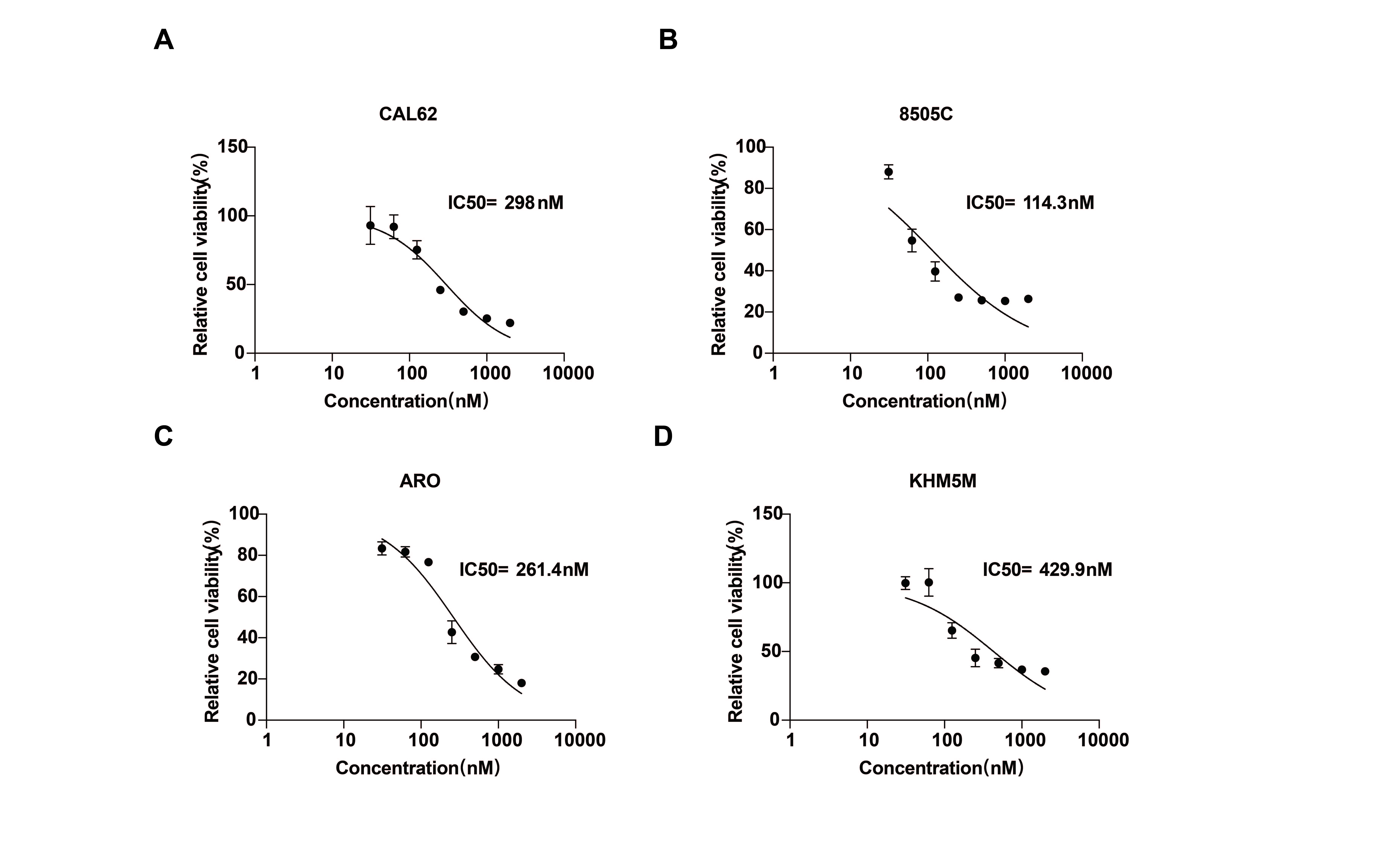
**

**Figure S1. Dose-response curves of b-AP15 in different ATC cells.** **A.** CAL62 cells. **B.** 8505C cells. **C.** ARO cells. **D.** KHM5M cells. Each panel shows the relative cell viability at various concentrations of b-AP15. The experiment was repeated three times.


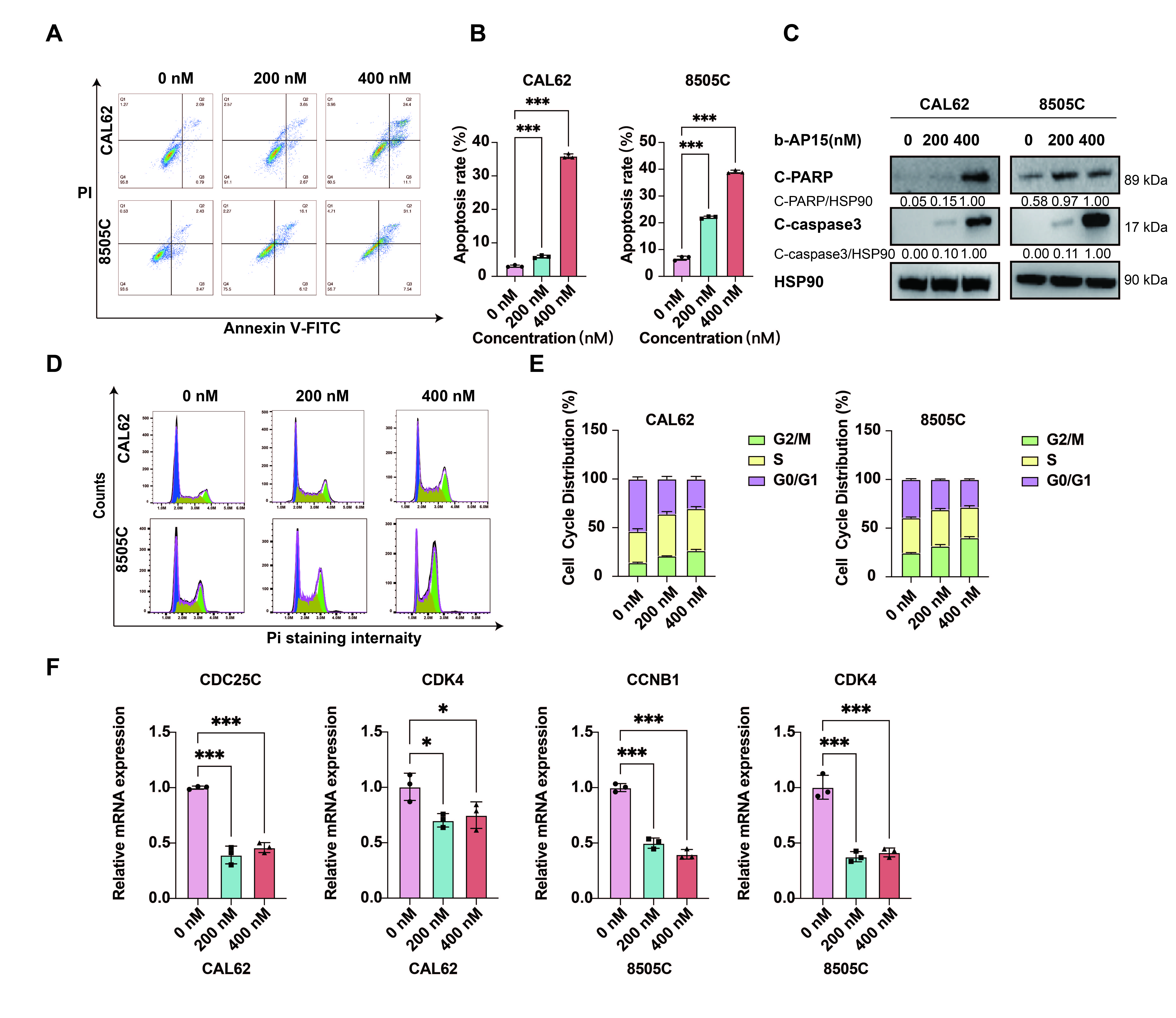


**Figure S2. Effects of b-AP15 on apoptosis and cell cycle in ATC cells.** **A.** Flow cytometry analysis of apoptosis in CAL62 and 8505C cells treated with 0 nM, 200 nM, and 400 nM b-AP15. **B.** Quantification of apoptosis rates in CAL62 and 8505C cells. The experiment was repeated three times. **C**. Western blotting analysis of C-PARP, C-caspase3, and HSP90 in CAL62 and 8505C cells. **D.** Flow cytometry analysis of cell cycle distribution in CAL62 and 8505C cells treated with 0 nM, 200 nM, and 400 nM b-AP15. **E.** Quantification of cell cycle distribution in CAL62 and 8505C cells. The experiment was repeated three times. **F.** Relative mRNA expression of CDC25C, CDK4, and CCNB1 in CAL62 and 8505C cells.The experiment was repeated three times.


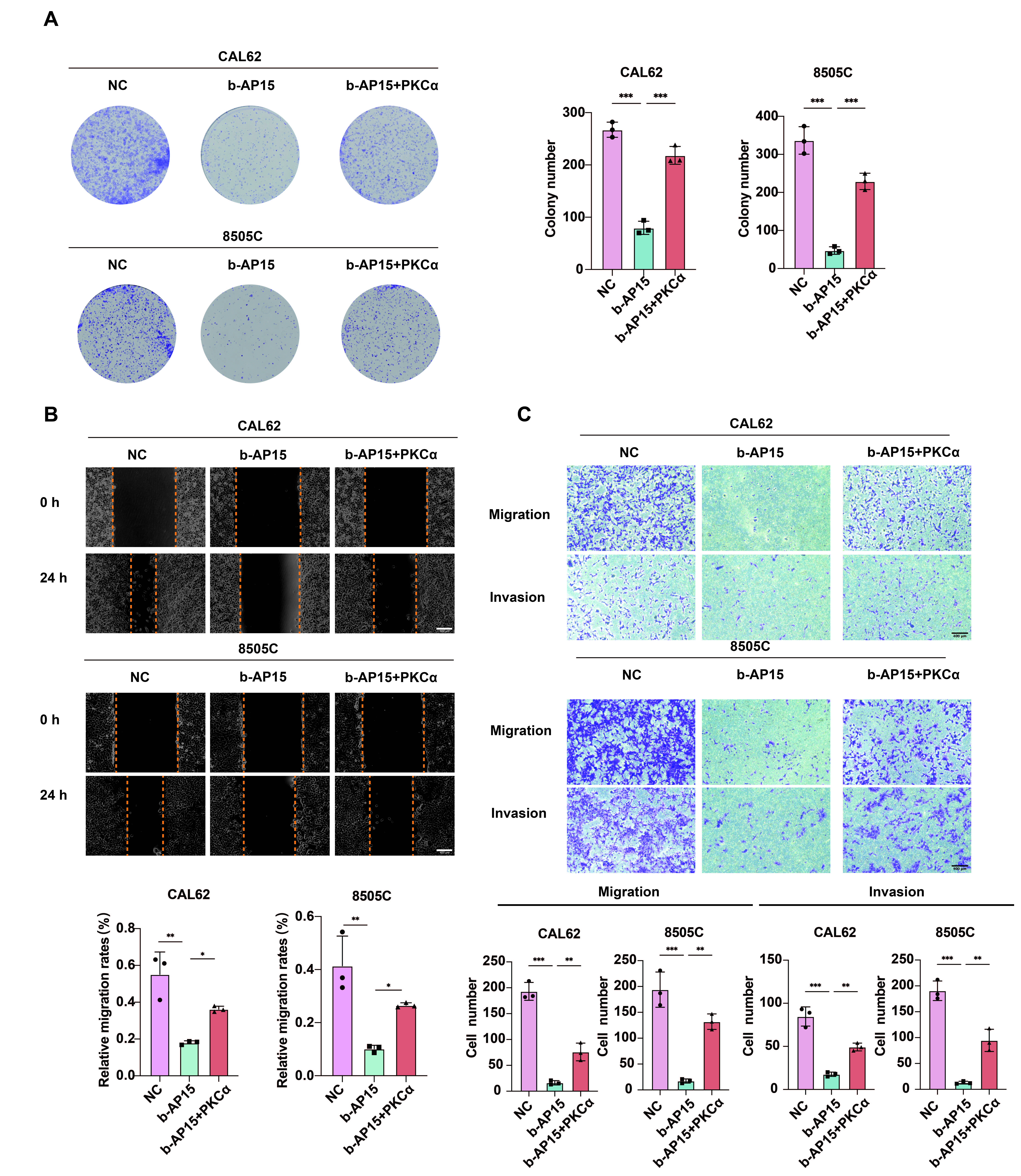


**Figure S3. b-AP15 promote the malignant phenotype of ATC cells by PKCα. A**. Colony formation assays were performed to assess the proliferation of cells treated with the b-AP15 and transfected with the PKCα plasmid. The experiment was repeated three times. **B**. Wound healing assays were performed to evaluate the migration ability of CAL62 and 8505C cells treated with the b-AP15 inhibitor and transfected with the PKCα plasmid at 0 and 24 hours. Scale bar, 400 μm. The experiment was repeated three times. **C**. Transwell assays were carried out to demonstrate the effects of the b-AP15 inhibitor and PKCα plasmid on the migration and invasion of CAL62 and 8505C cells. Scale bar, 400 μm. The experiment was repeated three times.





**Figure S4.** Immunofluorescence staining of p-p65 and PKCα in tumor tissues from nude mice in control and b-AP15 groups. Scale bar, 20μm.

**Table S1** List of siRNA sequence

| **Genes** | **siRNA Sequence** | |
| --- | --- | --- |
| USP14 | Sense | GCAGCCAAAUACAAGUGACAA |
|  | Antisense | UUGUCACUUGUAUUUGGCUGC |
| UCHL5 | Sense | UCCCGACUUGACACGAUAUUU |
|  | Antisense | AAAUAUCGUGUCAAGUCGGGA |
| PKCα | Sense #1 | CCCGUCUUAACACCACCUGAU |
|  | Antisense #1 | AUCAGGUGGUGUUAAGACGGG |
|  | Sense #2 | GCUGUACUUCGUCAUGGAAUA |
|  | Antisense #2 | UAUUCCAUGACGAAGUACAGC |

**Table S2** List of antibody used in this study

| **Antibody** | **Company** | **Catalog** | **Concentration** |
| --- | --- | --- | --- |
| USP14 | Proteintech | 14517-1-AP | 1:10000 |
| UCHL5 | Santa | sc-271002 | 1:500 |
| HSP90 | ABclonal | A5027 | 1:1000 |
| ZEB1 | Cell Signaling Technology | #3396 | 1:1000 |
| N-cadherin | Cell Signaling Technology | #13116 | 1:1000 |
| β-catenin | Cell Signaling Technology | #8480 | 1:1000 |
| Vimentin | Cell Signaling Technology | #5741 | 1:1000 |
| C-PARP | HUABIO | ET1608-10 | 1:1000 |
| C-caspase3 | Abcam | ab214430 | 1:5000 |
| PKCα | Proteintech | 21991-1-AP | 1:2000 |
| Flag | ABclonal | AE063 | 1:5000 |
| His | Proteintech | 66005-1-Ig | 1:5000 |
| GFP | Proteintech | 50430-2-AP | 1:1000 |
| GFP | Proteintech | 66002-1-Ig | 1:20000 |
| UB | Cell Signaling Technology | #20326 | 1:1000 |
| HA | Abclonal | AE008 | 1:5000 |
| P65 | Cell Signaling Technology | #8242 | 1:1000 |
| p-P65 | Cell Signaling Technology | #3033 | 1:1000 |
| IκBα | Cell Signaling Technology | #4814 | 1:1000 |
| p-IκBα | Cell Signaling Technology | #2859 | 1:1000 |

**Table S3** List of primer sequence for qRT-PCR

| **Genes** | **Primer Sequence** | |
| --- | --- | --- |
| USP14 | Forward | TGTGCCTGAACTCAAAGATGC |
|  | Reverse | ATATACTGCGCTGAAGCCATTT |
| UCHL5 | Forward | GAGTGGTGCCTCATGGAAAG |
|  | Reverse | CAAGTCGGGAGTCCTGAACC |
| GAPDH | Forward | GCACCGTCAAGGCTGAGAAC |
|  | Reverse | TGGTGAAGACGCCAGTGGA |
| ZEB1 | Forward | GATGATGAATGCGAGTCAGATGC |
|  | Reverse | ACAGCAGTGTCTTGTTGTTGT |
| CDH2 | Forward | AGCCAACCTTAACTGAGGAGT |
|  | Reverse | GGCAAGTTGATTGGAGGGATG |
| CTNNB1 | Forward | CATCTACACAGTTTGATGCTGCT |
|  | Reverse | GCAGTTTTGTCAGTTCAGGGA |
| VIM | Forward | AGTCCACTGAGTACCGGAGAC |
|  | Reverse | CATTTCACGCATCTGGCGTTC |
| CDH1 | Forward | ATTTTTCCCTCGACACCCGAT |
|  | Reverse | TCCCAGGCGTAGACCAAGA |
| SNAI2 | Forward | CGAACTGGACACACATACAGTG |
|  | Reverse | CTGAGGATCTCTGGTTGTGGT |
| CDK4 | Forward | ATGGCTACCTCTCGATATGAGC |
|  | Reverse | CATTGGGGACTCTCACACTCT |
| CCNB1 | Forward | TTGGGGACATTGGTAACAAAGTC |
|  | Reverse | ATAGGCTCAGGCGAAAGTTTTT |
| CDC25C | Forward | AAGTGGCCTATATCGCTCCC |
|  | Reverse | CCCTGGTTAGAATCTTCCTCCA |
| MYC | Forward | GTCAAGAGGCGAACACACAAC |
|  | Reverse | TTGGACGGACAGGATGTATGC |
| BCL2L1 | Forward | GAGCTGGTGGTTGACTTTCTC |
|  | Reverse | TCCATCTCCGATTCAGTCCCT |

**Table S4** List of differentially expressed proteins from proteomics analysis

| **Accession** | **Gene Name** | **b-ap15/Control** | **t test p value** |
| --- | --- | --- | --- |
| Q6ZP01 | RBM44 | 2.27119407 | 0.002407072 |
| Q86SR1 | GALNT10 | 2.219346035 | 0.011957756 |
| Q96T21 | SECISBP2 | 2.18663097 | 0.004869936 |
| Q9UL16 | CFAP45 | 2.181608868 | 0.002248536 |
| Q8N122 | RPTOR | 2.14958766 | 0.01125014 |
| P08F94 | PKHD1 | 2.097152672 | 0.006676493 |
| P19013 | KRT4 | 2.08871742 | 0.022576545 |
| Q9ULD2 | MTUS1 | 2.038030127 | 0.007888768 |
| Q9UBK9 | UXT | 2.03595387 | 0.007489682 |
| P21439 | ABCB4 | 2.011658876 | 5.51353E-05 |
| Q9NZJ0 | DTL | 1.998986241 | 0.006392832 |
| P24043 | LAMA2 | 1.98507101 | 0.048039641 |
| Q9Y255 | PRELID1 | 1.983449298 | 0.000944449 |
| P04731 | MT1A | 1.939139008 | 0.014034519 |
| O15169 | AXIN1 | 1.89044866 | 0.004290095 |
| P04732 | MT1E | 1.863913338 | 0.015737456 |
| P0CB47 | UBTFL1 | 1.854738769 | 0.003474291 |
| P30825 | SLC7A1 | 1.8409021 | 0.040917089 |
| Q9UDY6 | TRIM10 | 1.81443795 | 0.025831974 |
| Q643R3 | LPCAT4 | 1.805250695 | 0.031077404 |
| B7ZAP0 | RABGAP1L | 1.736002258 | 0.004465608 |
| P09601 | HMOX1 | 1.715227227 | 0.025508844 |
| P02795 | MT2A | 1.712036152 | 0.03112674 |
| O00488 | ZNF593 | 1.678407162 | 0.002337743 |
| Q96DE5 | ANAPC16 | 1.668719692 | 0.019860596 |
| Q9H4I3 | TRABD | 1.650715015 | 0.003756334 |
| P24557 | TBXAS1 | 1.621485162 | 0.011064042 |
| O95365 | ZBTB7A | 1.618751125 | 0.039655167 |
| Q92545 | TMEM131 | 1.616652087 | 0.005028878 |
| Q96N64 | PWWP2A | 1.616454402 | 0.045112964 |
| Q9H8G2 | CAAP1 | 1.615386717 | 0.001591042 |
| P53539 | FOSB | 1.601566926 | 0.025110391 |
| P00441 | SOD1 | 1.591489815 | 0.002229987 |
| Q8N5U6 | RNF10 | 1.58490219 | 0.032562772 |
| P69905 | HBA2 | 1.577842465 | 0.038212565 |
| Q2KHR3 | QSER1 | 1.564331993 | 0.034126243 |
| Q6V0I7 | FAT4 | 1.560545216 | 0.006163304 |
| Q92623 | TTC9 | 1.529047444 | 0.010436573 |
| Q9HC77 | CENPJ | 1.526085304 | 0.003184897 |
| Q9UDY4 | DNAJB4 | 1.51403501 | 0.004109623 |
| Q5JSZ5 | PRRC2B | 1.509632534 | 0.002025114 |
| Q14517 | FAT1 | 1.506743185 | 0.004552051 |
| Q13542 | EIF4EBP2 | 1.502967628 | 0.010991638 |
| Q9UGM3 | DMBT1 | 1.493541513 | 0.031060027 |
| O15084 | ANKRD28 | 1.484134184 | 0.005288619 |
| O60925 | PFDN1 | 1.481099104 | 0.011308669 |
| O95997 | PTTG1 | 1.473637178 | 0.03623434 |
| Q6IA69 | NADSYN1 | 1.472425051 | 0.008882616 |
| P02042 | HBD | 1.47036649 | 0.016106422 |
| Q16661 | GUCA2B | 1.463169206 | 0.02313719 |
| P32926 | DSG3 | 1.458036665 | 0.009530535 |
| P31431 | SDC4 | 1.447107801 | 0.000927131 |
| Q8NI22 | MCFD2 | 1.446808447 | 0.000273959 |
| Q9Y2K5 | R3HDM2 | 1.44298947 | 0.000608937 |
| Q6NT76 | HMBOX1 | 1.4419659 | 0.018739051 |
| Q9UJY4 | GGA2 | 1.441917023 | 0.008777599 |
| Q9UHF7 | TRPS1 | 1.438977357 | 0.012564601 |
| Q9P281 | BAHCC1 | 1.427092065 | 0.002484333 |
| Q9P2H5 | USP35 | 1.426424159 | 0.000370518 |
| P34931 | HSPA1L | 1.425782085 | 0.004713871 |
| P20338 | RAB4A | 1.421400708 | 0.017222163 |
| P57768 | SNX16 | 1.421144427 | 0.000654032 |
| Q16539 | MAPK14 | 1.419640886 | 0.042628427 |
| P04733 | MT1F | 1.419325549 | 0.04107155 |
| Q5KU26 | COLEC12 | 1.41905839 | 0.0165489 |
| Q9C037 | TRIM4 | 1.393977965 | 0.012419402 |
| Q0VDD7 | BRME1 | 1.388239825 | 0.000948197 |
| P17275 | JUNB | 1.387375598 | 0.010048007 |
| Q9C0B0 | UNK | 1.377666188 | 0.041916184 |
| Q9H0J9 | PARP12 | 1.374436662 | 0.001877059 |
| P46019 | PHKA2 | 1.368965473 | 0.027953797 |
| Q9Y6N6 | LAMC3 | 1.362448595 | 0.009772804 |
| Q8NC44 | RETREG2 | 1.360307147 | 0.032743769 |
| Q86VE9 | SERINC5 | 1.349190957 | 0.014181672 |
| Q8IV50 | LYSMD2 | 1.345965083 | 0.001232307 |
| Q96M96 | FGD4 | 1.342184288 | 0.035120314 |
| Q99622 | C12orf57 | 1.3414797 | 0.007526187 |
| Q9Y291 | MRPS33 | 1.341467901 | 0.020131772 |
| Q9BU89 | DOHH | 1.340728811 | 0.010200037 |
| Q9BRK5 | SDF4 | 1.33942722 | 0.002897069 |
| Q9UJT0 | TUBE1 | 1.336225827 | 0.003755306 |
| O95628 | CNOT4 | 1.333062674 | 0.011939431 |
| Q9Y4D8 | HECTD4 | 1.329541161 | 0.00817669 |
| O94886 | TMEM63A | 1.326680844 | 0.044962093 |
| Q9H2G4 | TSPYL2 | 1.322941136 | 0.008115148 |
| P52926 | HMGA2 | 1.315862059 | 0.045341347 |
| Q9NRY2 | INIP | 1.303087657 | 0.029861274 |
| Q9BT81 | SOX7 | 1.296864876 | 0.001480953 |
| P51957 | NEK4 | 1.296432758 | 0.010319079 |
| Q9BZQ6 | EDEM3 | 1.296084424 | 0.003112828 |
| Q96JC9 | EAF1 | 1.291318104 | 0.007552216 |
| Q13501 | SQSTM1 | 1.291213564 | 0.003008406 |
| P01584 | IL1B | 1.287646297 | 0.003553118 |
| Q9NWA0 | MED9 | 1.285192283 | 0.000675808 |
| P14209 | CD99 | 1.28458196 | 0.006707235 |
| O43639 | NCK2 | 1.280652094 | 0.003974464 |
| P17302 | GJA1 | 1.279272283 | 0.013960271 |
| Q9ULJ7 | ANKRD50 | 1.277712224 | 0.036075752 |
| Q96EN8 | MOCOS | 1.277595284 | 0.004392272 |
| Q8NG68 | TTL | 1.276736646 | 0.004312481 |
| Q9P0S2 | COX16 | 1.27591643 | 0.002942142 |
| Q92685 | ALG3 | 1.274023782 | 0.030598966 |
| Q8N6N3 | C1orf52 | 1.273247933 | 0.001963262 |
| Q6WKZ4 | RAB11FIP1 | 1.268026655 | 0.000803909 |
| Q9BU61 | NDUFAF3 | 1.266893813 | 0.002527431 |
| Q9BTE7 | DCUN1D5 | 1.263022024 | 0.047749534 |
| Q9Y3B9 | RRP15 | 1.261367707 | 0.00839037 |
| Q9UEG4 | ZNF629 | 1.25989409 | 0.044116894 |
| P05091 | ALDH2 | 1.258807846 | 0.002115444 |
| Q9NX08 | COMMD8 | 1.255886269 | 0.034284622 |
| Q9NVG8 | TBC1D13 | 1.255854779 | 0.00413039 |
| Q96E52 | OMA1 | 1.253658165 | 0.025389842 |
| Q9UQ88 | CDK11A | 1.250801995 | 0.012732375 |
| A0A0U1RRE5 | NBDY | 1.250418045 | 0.00013424 |
| O75348 | ATP6V1G1 | 1.250282133 | 0.014174528 |
| Q9H2K0 | MTIF3 | 1.249569003 | 0.001647301 |
| A6NKD9 | CCDC85C | 1.249567435 | 0.003504839 |
| Q86XE3 | MICU3 | 1.246984138 | 0.026849349 |
| Q92625 | ANKS1A | 1.246799458 | 0.000815451 |
| Q96MF7 | NSMCE2 | 1.241860047 | 0.006145357 |
| Q96RE7 | NACC1 | 1.241152913 | 0.018177951 |
| Q9BQI7 | PSD2 | 1.23904173 | 0.041501025 |
| Q96CN9 | GCC1 | 1.237328772 | 0.03103186 |
| P30533 | LRPAP1 | 1.235177844 | 0.01499485 |
| Q8NEZ2 | VPS37A | 1.23420524 | 0.025054083 |
| Q15834 | CCDC85B | 1.234071456 | 0.030143391 |
| Q53H47 | SETMAR | 1.233599538 | 0.01202121 |
| Q96N21 | TEPSIN | 1.232303662 | 0.016439069 |
| Q6P4R8 | NFRKB | 1.230513507 | 0.036135136 |
| Q9NR33 | POLE4 | 1.228863076 | 0.013663993 |
| Q02556 | IRF8 | 1.22866756 | 0.006294114 |
| Q9UGM1 | CHRNA9 | 1.22851297 | 0.021381518 |
| Q96BZ8 | LENG1 | 1.226538634 | 0.023837769 |
| Q9UGP4 | LIMD1 | 1.226365582 | 0.026918975 |
| Q9BUP3 | HTATIP2 | 1.225682514 | 0.049594089 |
| Q9BSQ5 | CCM2 | 1.224654941 | 0.012913736 |
| P19256 | CD58 | 1.22452215 | 0.00655002 |
| Q8WYQ5 | DGCR8 | 1.22332701 | 0.04976645 |
| Q5BKY9 | FAM133B | 1.223109843 | 0.001577838 |
| Q6P6B7 | ANKRD16 | 1.222997505 | 0.046681534 |
| Q15047 | SETDB1 | 1.222201524 | 0.043364515 |
| Q9NZ52 | GGA3 | 1.220546738 | 0.010518051 |
| P06730 | EIF4E | 1.220342675 | 0.021935103 |
| Q9BQE5 | APOL2 | 1.219927141 | 0.010646179 |
| Q16254 | E2F4 | 1.219783161 | 0.003576521 |
| Q9BRT6 | LLPH | 1.219167297 | 0.000327875 |
| P21127 | CDK11B | 1.216599894 | 0.047905632 |
| Q9Y3X0 | CCDC9 | 1.214263957 | 0.012915823 |
| Q9H3Q1 | CDC42EP4 | 1.211449495 | 0.00817249 |
| P58107 | EPPK1 | 1.210540572 | 0.037286011 |
| Q7Z5L9 | IRF2BP2 | 1.210191764 | 0.021954689 |
| O75528 | TADA3 | 1.210045694 | 0.043770959 |
| P62310 | LSM3 | 1.210015445 | 0.012924797 |
| O75190 | DNAJB6 | 1.209518354 | 0.043008356 |
| O00287 | RFXAP | 1.208682886 | 0.028114008 |
| Q9NX00 | TMEM160 | 1.20695414 | 0.03899752 |
| Q9H081 | MIS12 | 1.2053931 | 0.000299009 |
| Q53ET0 | CRTC2 | 1.203602376 | 0.049853221 |
| Q6GMV3 | PTRHD1 | 1.203418687 | 0.029234758 |
| O60739 | EIF1B | 1.203340144 | 0.016224726 |
| Q5H9L4 | TAF7L | 1.203008629 | 0.008515605 |
| Q96MW1 | CCDC43 | 1.202264165 | 0.016504466 |
| Q03468 | ERCC6 | 1.201574545 | 0.02046036 |
| Q8IYB9 | ZNF595 | 1.201454714 | 0.034781321 |
| P48509 | CD151 | 0.833196166 | 0.022889902 |
| Q9UJX5 | ANAPC4 | 0.83316247 | 0.043715778 |
| O95197 | RTN3 | 0.833074442 | 0.030368534 |
| Q9BSR8 | YIPF4 | 0.83152949 | 0.015328654 |
| Q96GN5 | CDCA7L | 0.830985736 | 0.029188567 |
| P04062 | GBA1 | 0.830554206 | 0.045396704 |
| O43674 | NDUFB5 | 0.830180136 | 0.020400132 |
| P10071 | GLI3 | 0.829919655 | 0.029784186 |
| O95149 | SNUPN | 0.829472975 | 0.012200031 |
| Q8IUH4 | ZDHHC13 | 0.82935766 | 0.007101792 |
| Q6AZY7 | SCARA3 | 0.829000919 | 0.049249305 |
| Q9BQP7 | MGME1 | 0.827541986 | 0.023109654 |
| Q9GZM5 | YIPF3 | 0.825517391 | 0.039316989 |
| Q9BZE4 | GTPBP4 | 0.824825623 | 0.049642034 |
| Q9UMR5 | PPT2 | 0.823992136 | 0.022684409 |
| Q9Y3U8 | RPL36 | 0.823066925 | 0.043619887 |
| Q93074 | MED12 | 0.821899662 | 0.01611873 |
| Q03113 | GNA12 | 0.821824362 | 0.020593394 |
| Q9UDW1 | UQCR10 | 0.821808274 | 0.030655284 |
| Q86VR2 | RETREG3 | 0.820987792 | 0.00987826 |
| Q8NE86 | MCU | 0.819252133 | 0.039416792 |
| P24941 | CDK2 | 0.819098331 | 0.041123723 |
| O15379 | HDAC3 | 0.819031611 | 0.033562598 |
| Q96BN2 | TADA1 | 0.818397579 | 0.010032445 |
| Q8TAP6 | CEP76 | 0.818388642 | 0.00572661 |
| P29323 | EPHB2 | 0.818140539 | 0.005011385 |
| O43688 | PLPP2 | 0.815731878 | 0.039422661 |
| Q9C0E8 | LNPK | 0.814376177 | 0.042944943 |
| Q14156 | EFR3A | 0.813818788 | 0.003804273 |
| O15270 | SPTLC2 | 0.81314441 | 0.027500012 |
| Q8IYB1 | MB21D2 | 0.812929102 | 0.023294877 |
| Q8TCE6 | DENND10 | 0.812731789 | 0.013999693 |
| P24311 | COX7B | 0.812345239 | 0.041925078 |
| P34896 | SHMT1 | 0.809773789 | 0.00334298 |
| P17252 | PRKCA | 0.808362548 | 0.049273884 |
| Q8IZD9 | DOCK3 | 0.807982286 | 0.034475433 |
| Q13613 | MTMR1 | 0.80764835 | 0.009513573 |
| Q8TBQ9 | TMEM167A | 0.80759089 | 0.02028889 |
| Q9Y251 | HPSE | 0.807122111 | 0.022775975 |
| A0JNW5 | BLTP3B | 0.807091015 | 0.018281214 |
| Q15646 | OASL | 0.80687258 | 0.00806014 |
| Q9NRG9 | AAAS | 0.806671232 | 0.018888743 |
| Q9NQH7 | XPNPEP3 | 0.806238651 | 0.036044673 |
| Q05639 | EEF1A2 | 0.805853369 | 0.027545439 |
| Q8IY95 | TMEM192 | 0.805525886 | 0.024000039 |
| P13051 | UNG | 0.805436153 | 0.005155272 |
| Q5VSL9 | STRIP1 | 0.802175031 | 0.046207531 |
| P22234 | PAICS | 0.801166958 | 0.033914478 |
| Q8NHQ9 | DDX55 | 0.801067242 | 0.014747479 |
| P11274 | BCR | 0.79979539 | 0.03124971 |
| Q9BXT2 | CACNG6 | 0.796666878 | 0.042588803 |
| O95864 | FADS2 | 0.795597366 | 0.033754218 |
| Q6YHU6 | THADA | 0.792479858 | 0.028183996 |
| Q9NS40 | KCNH7 | 0.788694459 | 0.046027413 |
| Q96BQ5 | CCDC127 | 0.78748498 | 0.037667121 |
| P06493 | CDK1 | 0.785902532 | 0.044876699 |
| Q9H0W8 | SMG9 | 0.785326575 | 0.013452665 |
| Q9NW08 | POLR3B | 0.783671609 | 0.002870216 |
| Q96LT4 | SAMD8 | 0.783470512 | 0.045122264 |
| Q6AI08 | HEATR6 | 0.779643732 | 0.000330087 |
| P49768 | PSEN1 | 0.778263562 | 0.03027284 |
| P46977 | STT3A | 0.777595754 | 0.033128065 |
| Q86YT6 | MIB1 | 0.77678041 | 0.043374129 |
| Q9Y2P8 | RCL1 | 0.776246803 | 0.017478695 |
| Q9NUN5 | LMBRD1 | 0.772666577 | 0.021977293 |
| Q9P0J0 | NDUFA13 | 0.769866239 | 0.021108674 |
| Q9Y4K0 | LOXL2 | 0.769469446 | 0.009398742 |
| Q15386 | UBE3C | 0.76926644 | 0.013483814 |
| Q13315 | ATM | 0.766985705 | 0.036766959 |
| Q96F07 | CYFIP2 | 0.762966378 | 0.01545738 |
| Q5RI15 | COX20 | 0.757333803 | 0.028057334 |
| Q8IV38 | ANKMY2 | 0.757001137 | 0.014678556 |
| Q86VU5 | COMTD1 | 0.756064219 | 0.019058327 |
| O60831 | PRAF2 | 0.756014725 | 0.010115796 |
| Q9NRX5 | SERINC1 | 0.755340193 | 0.01759157 |
| Q9BRX8 | PRXL2A | 0.753581248 | 0.010223318 |
| Q8IXB1 | DNAJC10 | 0.743755645 | 0.008890447 |
| Q969Z3 | MTARC2 | 0.741724695 | 0.045081231 |
| Q9Y2U5 | MAP3K2 | 0.725371647 | 0.016639106 |
| Q12905 | ILF2 | 0.719098628 | 0.039058005 |
| Q9Y324 | FCF1 | 0.718078794 | 0.018016928 |
| Q86X27 | RALGPS2 | 0.691256398 | 0.034190648 |
| Q5TEC6 | H3-7 | 0.684989956 | 0.007701538 |
| P51530 | DNA2 | 0.673415546 | 0.00769854 |
| P35790 | CHKA | 0.661890714 | 0.006326523 |
| Q92604 | LPGAT1 | 0.644043667 | 0.002281568 |
| O75508 | CLDN11 | 0.633705193 | 0.000397511 |
| Q9Y5Z7 | HCFC2 | 0.62397652 | 0.030203302 |
| Q04756 | HGFAC | 0.587070644 | 0.001566414 |

**Table S5** Positive ratio of immunoreactivity intensity for USP14 and UCHL5

|  | **USP14** | | | | **UCHL5** | | |
| --- | --- | --- | --- | --- | --- | --- | --- |
| **No.** | **NT** | **PTC** | **ATC** | **NT** | | **PTC** | **ATC** |
| **1** | - | - | ++ | - | | - | +++ |
| **2** | - | + | ++ | - | | - | +++ |
| **3** | - | + | ++ | - | | + | +++ |
| **4** | - | + | ++ | - | | + | + |
| **5** | - | + | ++ | - | | + | + |
| **6** | - | + |  | - | | + |  |
| **7** | - | + |  | - | | + |  |
| **8** | - | ++ |  | - | | + |  |
| **9** | - |  |  | - | |  |  |
| **10** | - |  |  | - | |  |  |
| **11** | - |  |  | - | |  |  |
| **Positive Ratio** | 0/11 | 7/8 | 5/5 | 0/11 | | 6/8 | 5/5 |

Note: “-” indicates negative; “+” indicates weak positive; “++” indicates moderate positive; “+++” indicates strong positive.
