# Supplementary figures and images for: USP14 and UCHL5 synergistically deubiquitinate PKCα and translocate NF-κB to promote the progression of anaplastic thyroid cancer
Source: Cell Death Dis. 2025 Aug 13;16(1):617. doi: 10.1038/s41419-025-07890-9 (PMC12350928; doi:10.1038/s41419-025-07890-9)

Figure 1

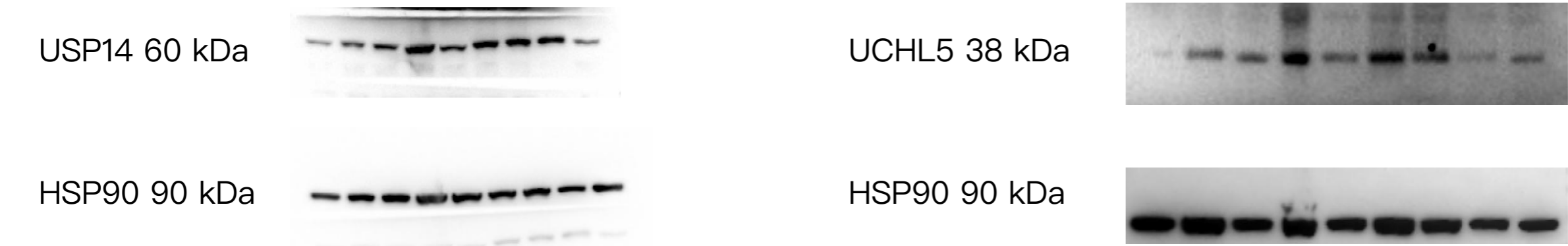

Figure 2

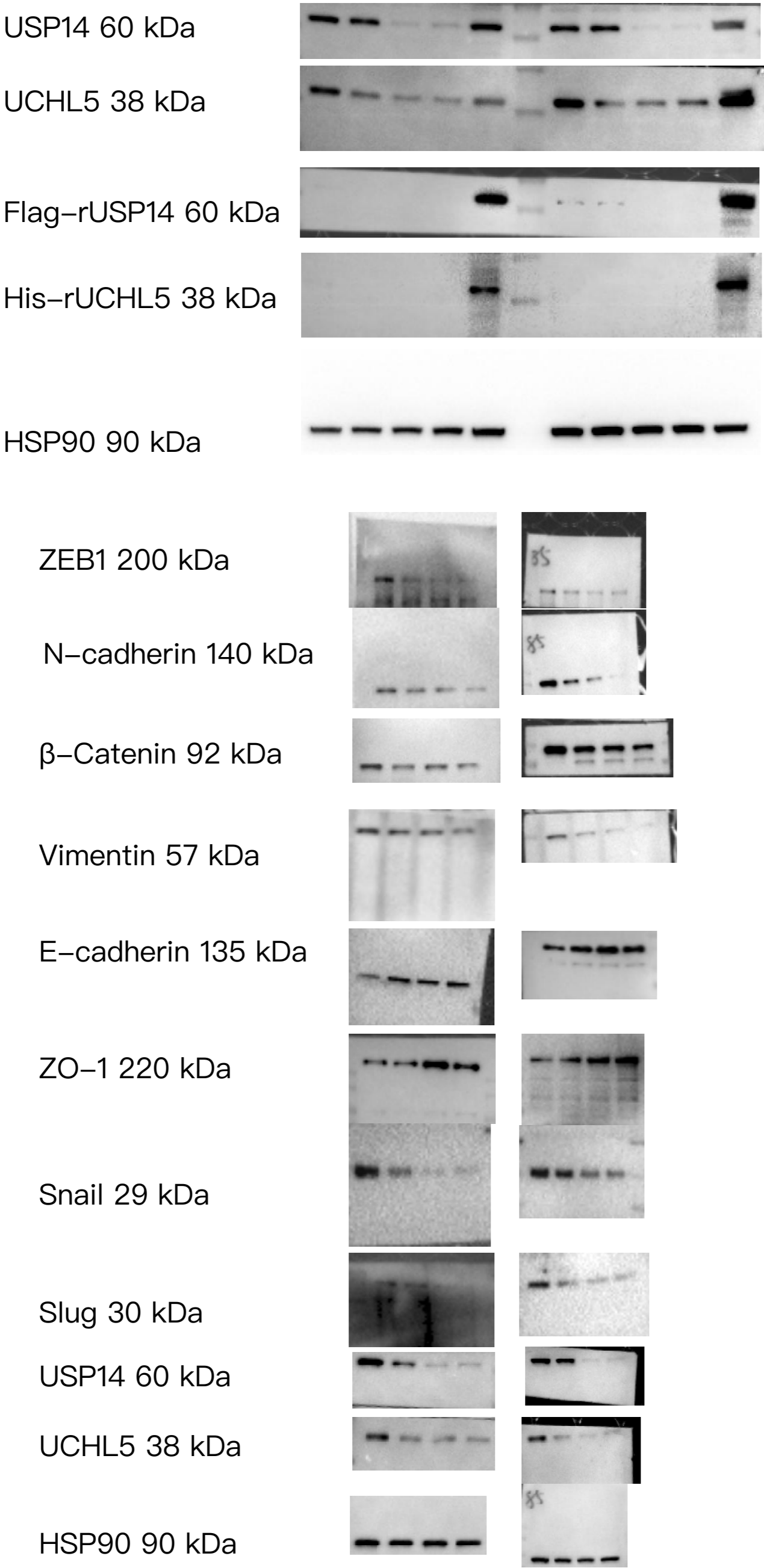

Figure 3

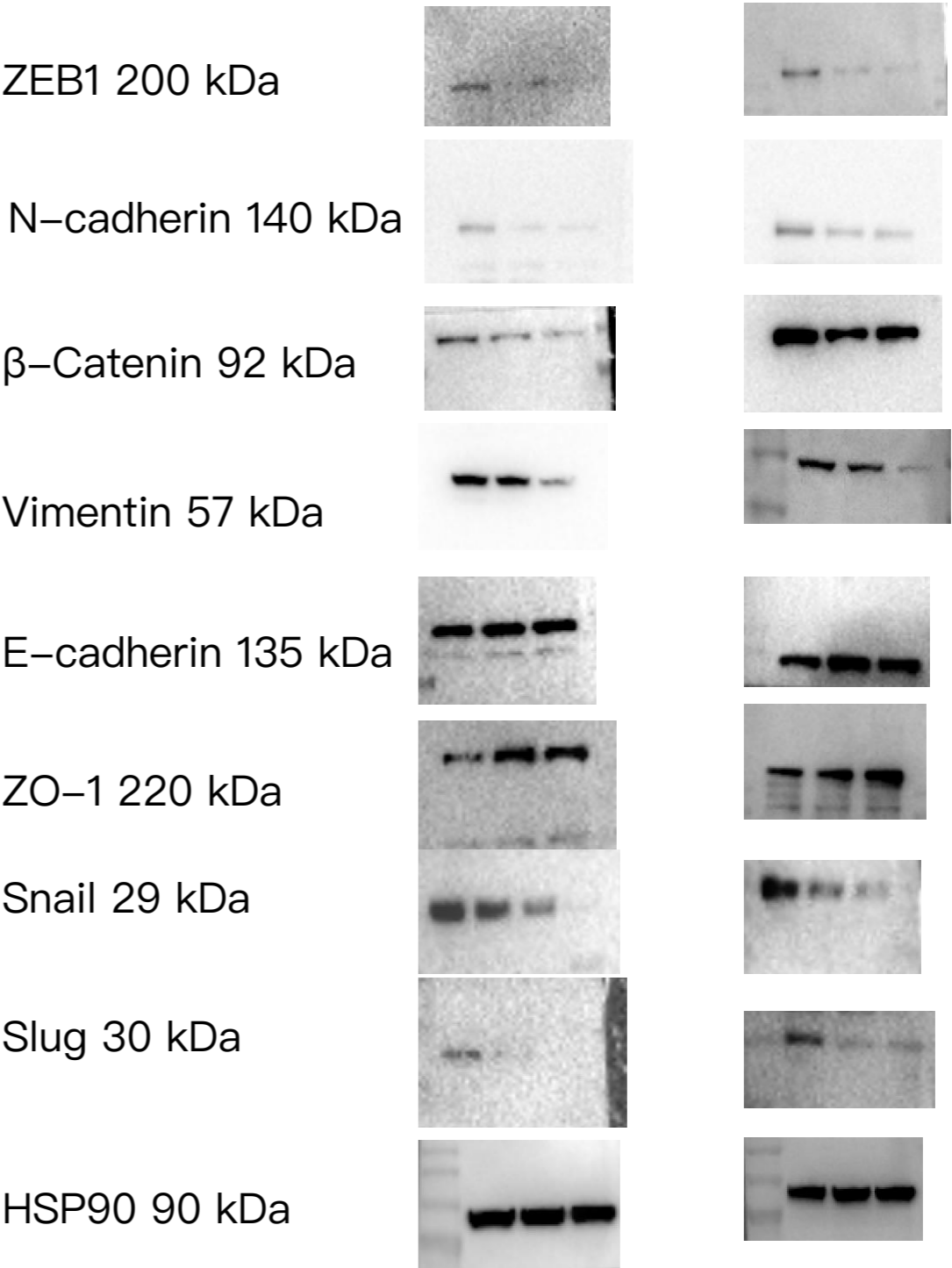

Figure 4

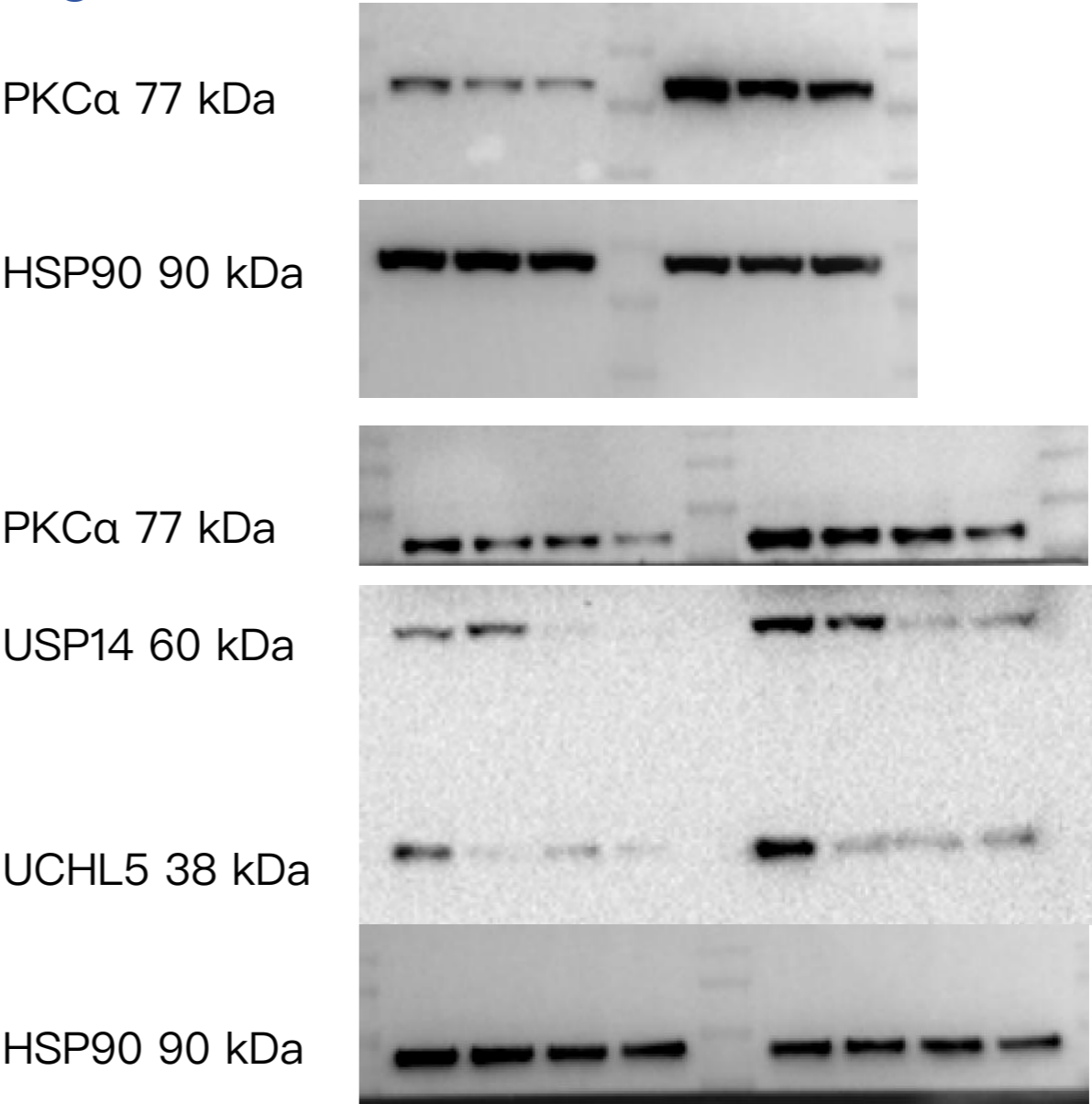

Figure 5

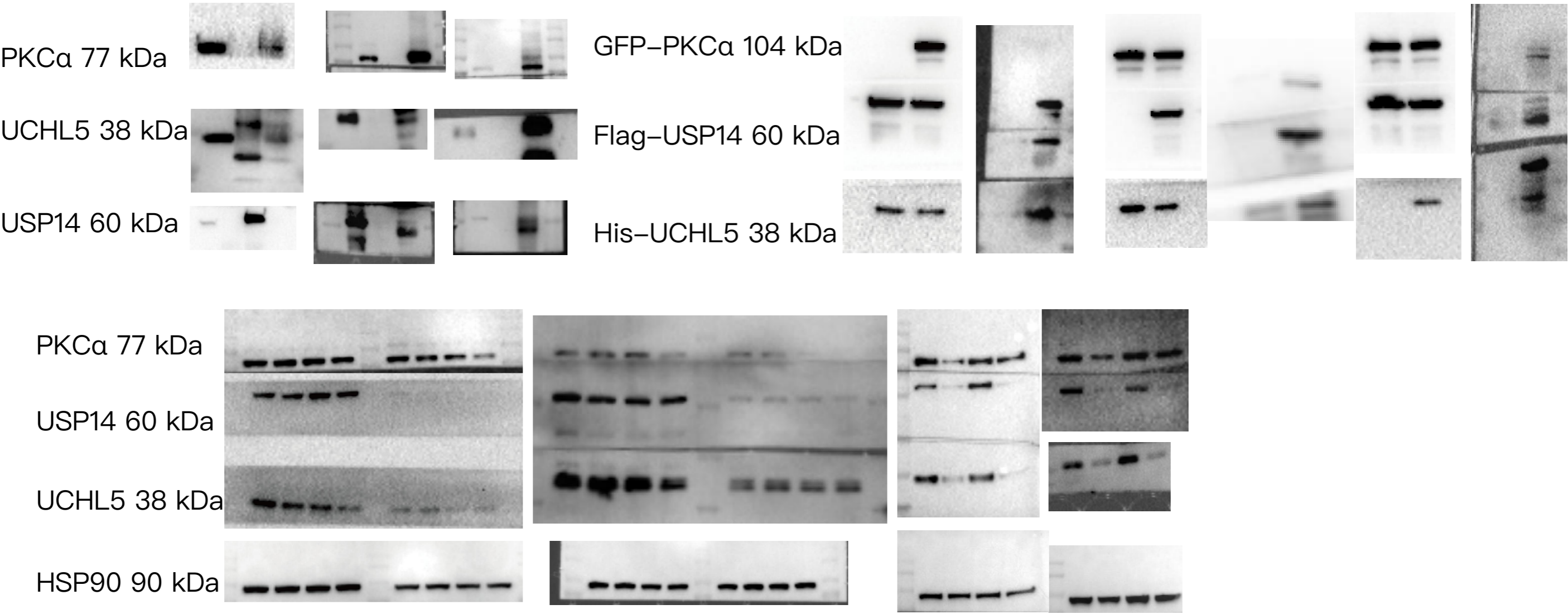

Figure 6

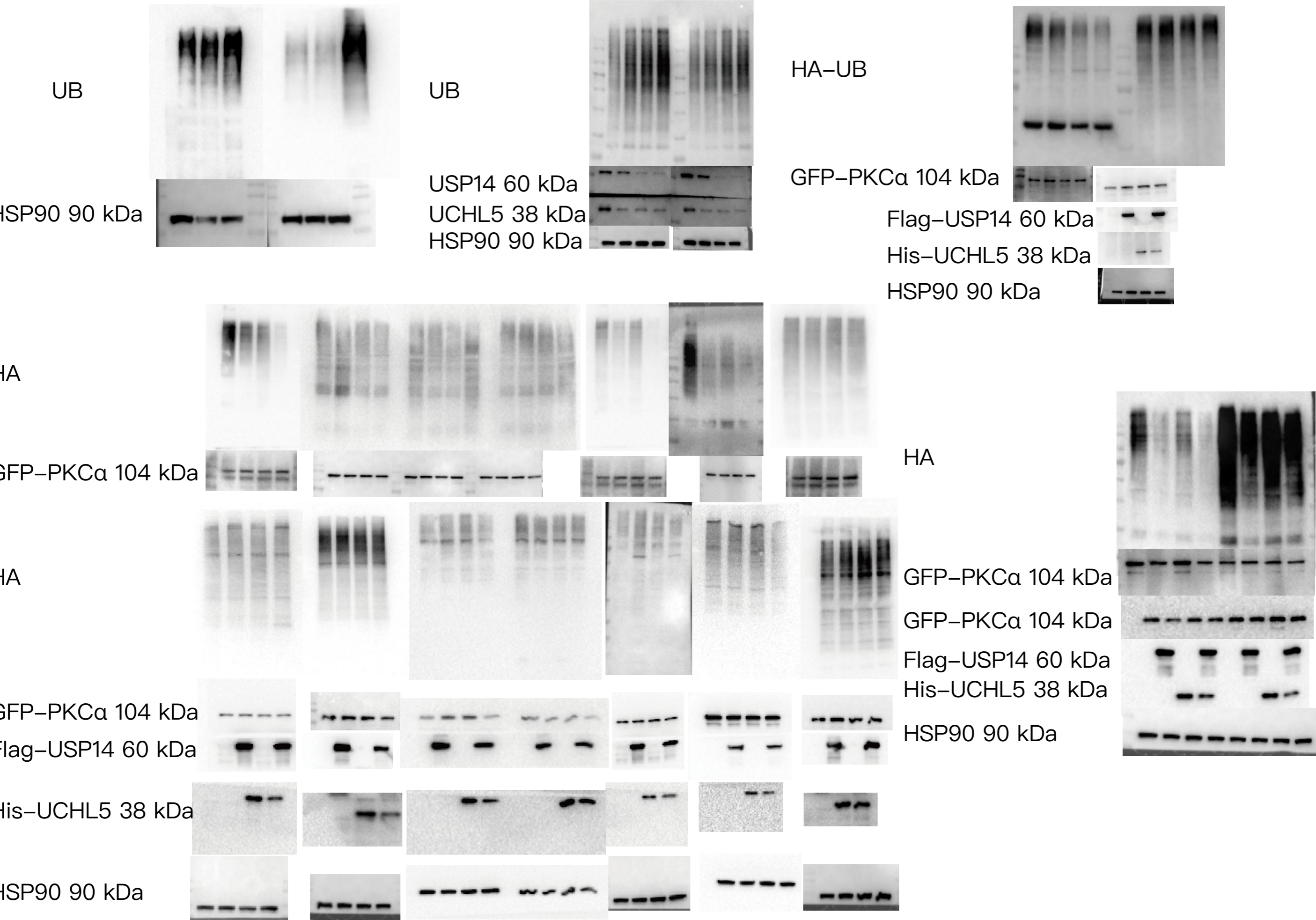

Figure 7

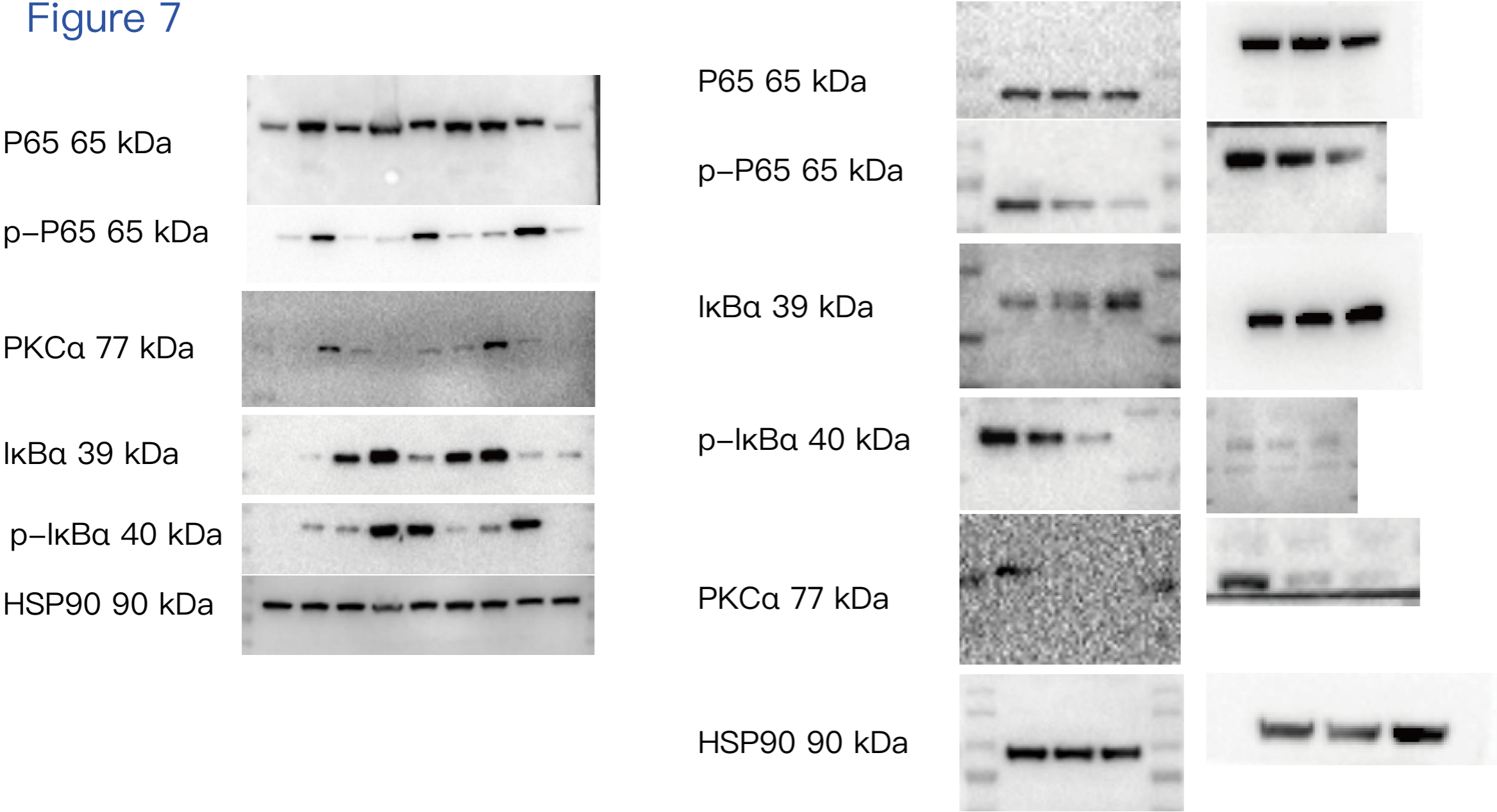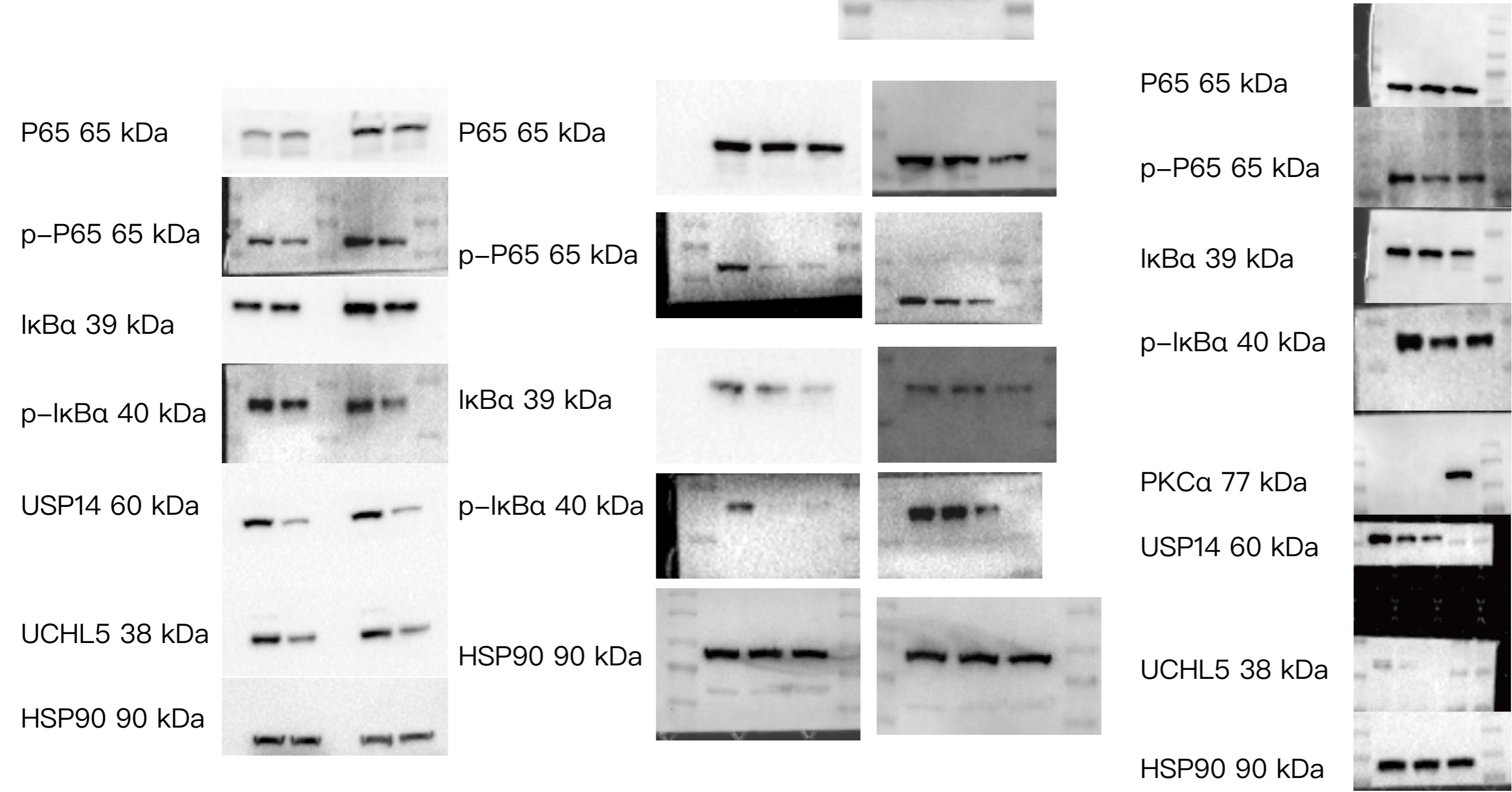

Figure S2

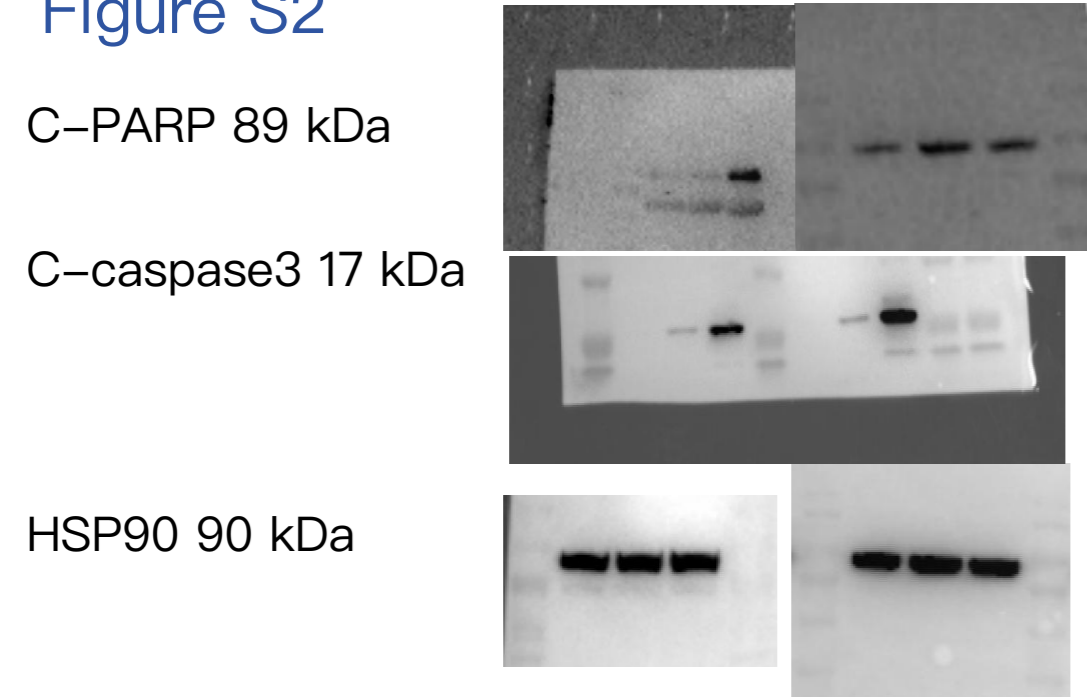

Supplement: Supplementary file 2 — Original WB data [file 41419_2025_7890_MOESM2_ESM.pdf]
